# PINK1 Is Selectively Stabilized on Impaired Mitochondria to Activate Parkin

**DOI:** 10.1371/journal.pbio.1000298

**Published:** 2010-01-26

**Authors:** Derek P. Narendra, Seok Min Jin, Atsushi Tanaka, Der-Fen Suen, Clement A. Gautier, Jie Shen, Mark R. Cookson, Richard J. Youle

**Affiliations:** 1 Biochemistry Section, Surgical Neurology Branch, National Institute of Neurological Disorders and Stroke, National Institutes of Health, Bethesda, Maryland, United States of America; 2 Center for Neurologic Diseases, Brigham and Women's Hospital, Program in Neuroscience, Harvard Medical School, Boston, Massachusetts, United States of America; 3 Cell Biology and Gene Expression Unit, Laboratory of Neurogenetics, National Institute on Aging, Bethesda, Maryland, United States of America; St. Jude Children's Research Hospital, United States of America

## Abstract

Loss-of-function mutations in PINK1 and Parkin cause parkinsonism in humans and mitochondrial dysfunction in model organisms. Parkin is selectively recruited from the cytosol to damaged mitochondria to trigger their autophagy. How Parkin recognizes damaged mitochondria, however, is unknown. Here, we show that expression of PINK1 on individual mitochondria is regulated by voltage-dependent proteolysis to maintain low levels of PINK1 on healthy, polarized mitochondria, while facilitating the rapid accumulation of PINK1 on mitochondria that sustain damage. PINK1 accumulation on mitochondria is both necessary and sufficient for Parkin recruitment to mitochondria, and disease-causing mutations in PINK1 and Parkin disrupt Parkin recruitment and Parkin-induced mitophagy at distinct steps. These findings provide a biochemical explanation for the genetic epistasis between PINK1 and Parkin in *Drosophila melanogaster*. In addition, they support a novel model for the negative selection of damaged mitochondria, in which PINK1 signals mitochondrial dysfunction to Parkin, and Parkin promotes their elimination.

## Abstract 2

Mutations in PINK1 or Parkin lead to familial parkinsonism. The authors suggest that PINK1 and Parkin form a pathway that senses damaged mitochondria and selectively targets them for degradation.

## Author Summary

Mutations in the PINK1 or Parkin genes lead to an inherited form of Parkinson disease. Understanding how the products of these genes work may give us insights into what goes wrong in these patients and in Parkinson disease more generally. Previous studies in flies and mice, and in human cells suggest that PINK1 and Parkin are part of a common pathway that protects against damaged mitochondria; these organelles power the cell when healthy but can produce harmful reactive oxygen species when damaged. Exactly how PINK1 and Parkin work together to protect against damaged mitochondria is unclear. The findings we report in this paper suggest a new model in which PINK1 and Parkin together sense mitochondria in distress and selectively target them for degradation. In this pathway, PINK1 acts as a flag that accumulates on dysfunctional mitochondria and then signals to Parkin, which tags these mitochondria for destruction. Since disease-causing mutations in PINK1 or Parkin disrupt this pathway, patients with these mutations may not be able to clean up their damaged mitochondria, leading to the neuronal damage typical of parkinsonism.

## Introduction

Parkinson disease is a common neurodegenerative disorder with no disease-modifying therapy presently available for its treatment [1]. Study of recessive forms of familial Parkinson disease, such as those resulting from mutations in the E3 ubiquitin ligase Parkin (GeneID: 5071) or the mitochondrial kinase PINK1 (GeneID: 65018), may reveal disease mechanisms important to the development of disease in these families as well as those suffering from sporadic Parkinson disease.

Although the cause of sporadic Parkinson disease is likely complex, several lines of evidence link mitochondrial dysfunction to its pathogenesis. Mitochondria within the substantia nigra (SN), a midbrain region that is preferentially affected in Parkinson disease, have a higher somatic mitochondrial DNA (mtDNA) mutation rate than all other regions of the brain examined [2]. Increased mitochondrial damage in the SN, particularly to mtDNA, has been associated with sporadic Parkinson disease [3]–[5], and mitochondrial dysfunction is sufficient to cause parkinsonism in patients with rare multiple mtDNA deletion syndromes and in animal models with decreased mtDNA expression [6]–[8]. In addition, toxins such as MPTP and rotenone, which are believed to increase reactive oxygen species from complex I of the electron transport chain, can induce a parkinsonian syndrome in humans and animal models [9],[10]. Since neurons in the SN are postmitotic, any mitochondrial damage they acquire could accumulate over an organism's lifetime, leading to progressive mitochondrial dysfunction—including increased oxidative stress, decreased calcium buffering capacity, loss of ATP, and, eventually, cell death—unless quality control processes eliminate the damaged mitochondria.

Recent studies have linked Parkin and PINK1 in a pathway critical for the maintenance of mitochondrial integrity and function. Loss of either protein in *Drosophila* results in a similar phenotype, with mitochondrial damage preceding muscle degeneration, as well as disrupted spermatogenesis and death of dopaminergic neurons [11]–[15]. Interestingly, overexpression of Parkin can partially compensate for PINK1 loss, but PINK1 overexpression cannot compensate for Parkin loss, suggesting that PINK1 functions upstream of Parkin in a common pathway. Additionally, mice null for either Parkin or PINK1 exhibit increased oxidative damage and decreased mitochondrial function in the striatium (which receives projections from dopaminergic neurons) [16],[17]; and primary cells from patients with loss-of-function mutations in Parkin or PINK1 have similar abnormalities [18]–[20]. Together these findings suggest that Parkin and PINK1 may function in an evolutionarily conserved pathway critical for the maintenance of mitochondrial integrity and function. We recently reported that Parkin is selectively recruited to dysfunctional mitochondria with low membrane potential and, subsequently, promotes their autophagic degradation [21]. This suggests that Parkin may limit mitochondrial damage by acting in a pathway that identifies and eliminates damaged mitochondria from the mitochondrial network. How mitochondrial dysfunction is signaled to Parkin, however, is unknown.

Here, we show that full-length PINK1 accumulates selectively on dysfunctional mitochondria, and that Parkin recruitment to depolarized mitochondria and subsequent Parkin-induced mitophagy are strictly dependent on PINK1's mitochondrial targeting signal and depolarization-induced accumulation. Together, these results strongly support a novel model for signaling between PINK1 and Parkin in response to mitochondrial damage. In this model, mitochondrial PINK1 is rapidly turned over on bioenergetically well-coupled mitochondria by proteolysis, but is selectively stabilized on mitochondria with low membrane potential. Selective accumulation of PINK1 on the impaired mitochondria recruits Parkin, and Parkin, in turn, induces the degradation of the damaged mitochondria. In this model, PINK1 and Parkin form a pathway for sensing and selectively eliminating damaged mitochondria from the mitochondrial network. Disease-causing mutations in PINK1 and/or Parkin disrupt this pathway at distinct steps, consistent with the pathway's importance for preventing early-onset parkinsonism.

## Results

### PINK1 Accumulates following Mitochondrial Depolarization

**Figure 1:**
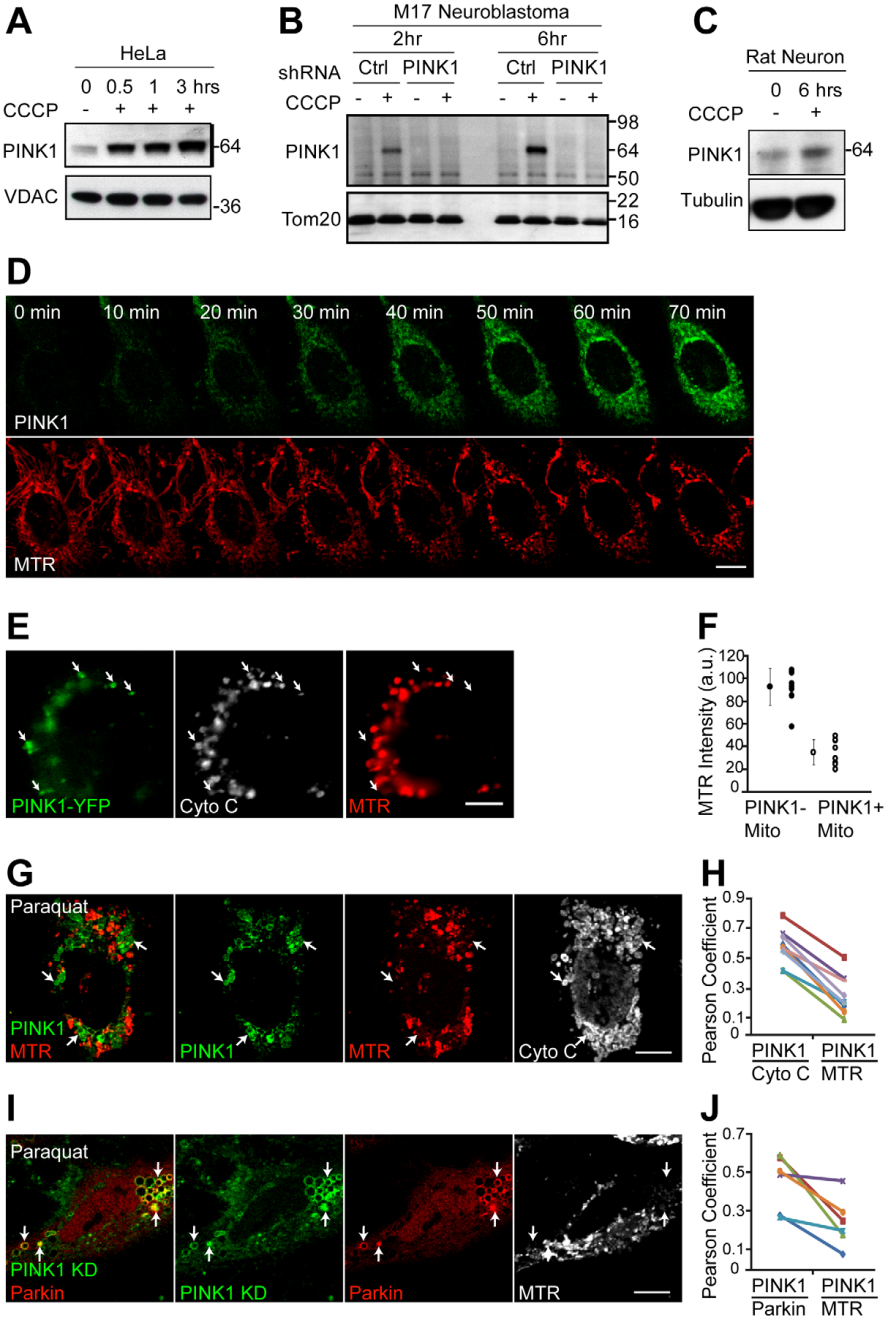
PINK1 selectively accumulates on depolarized mitochondria. (A) HeLa cells stably expressing YFP-Parkin were treated with 10 µM CCCP in serum at time point 0, fractionated, and carbonate extracted. The carbonate-extracted pellet, which is enriched in integral mitochondrial proteins, was run on SDS gels and immunoblotted for endogenous PINK1 and the mitochondrial protein VDAC. HeLa cells stably expressing YFP-Parkin were used in our initial experiments because it was unclear whether the stability of PINK1 would be affected by the absence of Parkin, as has been reported previously [29]. (B) M17 human neuroblastoma cells stably transduced with control shRNA or PINK1 shRNA were treated with 20 µM CCCP in serum and fractionated. The mitochondria-rich membrane fraction was run on SDS gels and immunoblotted as in (A). (C) E18 rat cortical neurons (7 d in vitro) were transfected with PINK1-V5. The next day the cells were treated with 1 µM CCCP for 6 h. Whole-cell lysates were run on SDS page gels and immunoblotted as in (A). (D) Live-cell imaging of HeLa cells transfected with PINK1-YFP (green) were treated with 10 µM CCCP in serum at time point 0. Mitochondria were labeled by pulsing with Mitotracker Red (MTR) (red) before depolarization with CCCP. (E) Mfn1/2 null MEFs transfected with PINK1-YFP (green). All mitochondria were stained with antibody against cytochrome c (Cyto C; white) and bioenergetically coupled mitochondria were stained by pulsing cells with Mitotracker Red (MTR) (red). (F) Average MTR intensity/pixel for PINK1-negative mitochondria (PINK1− Mito) and PINK1-positive mitochondria (PINK1+ Mito), respectively, was measured in eight or more cells in two independent experiments. Data from a representative experiment are shown. a.u., arbitrary units. (G) HeLa cells transfected with PINK1-YFP (green) and treated for 16 h with 2 mM paraquat. Cells were pulsed with MTR (red), fixed, and immunostained for cytochrome c (white). (H) The Pearson coefficient indexes between PINK1-YFP intensity and cytochrome c intensity, and PINK1-YFP intensity and MTR intensity were determined for eight or more cells in two independent experiments. Data from a representative experiment are shown. (I) HeLa cells were transfected with CFP-Parkin (red) and PINK1KD-YFP (green), which served as a reporter for endogenous PINK1 accumulation, and treated for 16 h with 2 mM paraquat. Cells were pulsed with MTR (white) and fixed. (J) The Pearson coefficient indexes between PINK1 KD-YFP intensity and CFP-Parkin intensity and PINK1 KD-YFP intensity and MTR intensity were determined for seven or more cells in two independent experiments. Data from a representative experiment are shown.

Parkin is selectively recruited to damaged mitochondria that have lost their membrane potential, but how Parkin distinguishes dysfunctional mitochondria with low membrane potential from healthy mitochondria is unknown. Since PINK1 is genetically upstream of Parkin, we tested whether PINK1's activity might be activated by mitochondrial depolarization. Remarkably, levels of endogenous mitochondrial PINK1 respond robustly to changes in mitochondrial membrane potential. When HeLa cells are treated with CCCP, which depolarizes mitochondria by increasing membrane permeability to H^+^, a large increase in endogenous full-length PINK1 (∼63 kDa) is seen beginning by 30 min and continuing for at least 3 h (Figure 1A). This ∼63-kDa band also increases in the mitochondria-rich membrane fraction following treatment with valinomycin, which, unlike CCCP, depolarizes mitochondria by permeabilizing the membrane to K^+^ (Figure S1A). By contrast, no band increases in the cytosolic fraction following depolarization with CCCP (Figure S1B).

To verify that the ∼63-kDa band is in fact PINK1, we immunoblotted for endogenous PINK1 in M17 cells stably transduced with control short hairpin RNA (shRNA) or PINK shRNA. We found that the ∼63-kDa band increases following CCCP treatment in control shRNA cells, but does not increase in the PINK1 shRNA cells, demonstrating that this ∼63-kDa band is endogenous PINK1 (Figure 1B). Similar results were found in PINK1^−/−^ cells transfected with PINK1-myc or left untransfected (Figure S1C). We also tested whether PINK1 similarly accumulates in primary rat cortical neurons following depolarization with CCCP. Although we (and others) failed to detect endogenous rat or mouse PINK1 with the available commercial antibodies ([22] and unpublished data), we observed PINK1-V5 increases in cortical neurons following treatment with 1 µM of CCCP for 6 h (Figure 1C). With CCCP treatment, PINK1 may accumulate more slowly in primary neurons than in HeLa cells, because, unlike HeLa cells [23], neurons rely almost exclusively on oxidative phosphorylation for ATP production [24].

To explore the kinetics of PINK1 accumulation at the single-cell level, we fused YFP to PINK1 and imaged cells live following depolarization with CCCP. Consistent with results obtained by Western blotting, we found that PINK1-YFP expression steadily increases from 1–5 min, when an increase is first detectable, until at least 70 min (Figure 1D and Video S1).

### PINK1 Accumulates Preferentially on Depolarized Mitochondria in a Single Cell

To examine the selectivity of PINK1 accumulation on uncoupled mitochondria within single cells, we first investigated its expression in mouse embryonic fibroblasts (MEFs) null for mitochondrial fusion proteins mitofusin-1 and mitofusin-2 (Mfn1/2). The Mfn1/2 null MEFs have a heterogeneous population of mitochondria, some of which are bioenergetically uncoupled and some of which are well coupled [25]. We found that, similar to YFP-Parkin [21], PINK1-YFP accumulates selectively on mitochondria with low membrane potential, demonstrating that PINK1 is selectively stabilized on the depolarized mitochondria within a bioenergetically diverse population of mitochondria (Figure 1E and 1F).

Treatment with paraquat, a pesticide that has been linked to Parkinsonism, also results in a heterogeneous population of mitochondria, likely due to stochastic damage of mitochondria by reactive oxygen species [26]. We treated HeLa cells overnight with a high dose of paraquat (2 mM). Similar to results with Parkin reported previously [21], we found that PINK1-YFP accumulates preferentially on damaged mitochondria with low membrane potential (Figure 1G). Although PINK1-YFP colocalizes with cytochrome c, which is present in all mitochondria (average Pearson coefficient = 0.58±0.11), PINK1-YFP does not colocalize with MTR (average Pearson coefficient = 0.26±0.13), which accumulates only in bioenergetically active mitochondria (*p*-value <0.001 for PINK1/cytochrome c vs. PINK1/MTR, paired Student *t*-test). These data suggest that PINK1-YFP accumulates selectively on depolarized mitochondria that have been damaged by oxidative stress (Figure 1H).

Next, we examined whether Parkin is recruited to the same depolarized mitochondria that accumulate PINK1 following treatment with paraquat. This relationship is difficult to test directly, because overexpression of PINK1 appears to accelerate the kinetics of Parkin recruitment to mitochondria (as shown later) [27], and so we used a kinase-deficient version of PINK1 (PINK1 KD) [28], as a reporter for endogenous PINK1 accumulation in the HeLa cells. We find PINK1 KD expression is regulated by mitochondrial voltage similarly to wild-type PINK1 (Figure S1D); but unlike wild-type PINK1, PINK1 KD does not enhance Parkin recruitment when overexpressed (as shown later). After treatment with paraquat overnight, we find that PINK1 KD accumulates selectively on depolarized mitochondria, in a pattern similar to wild-type PINK1 (Figure 1I). In addition, a substantial subset of the mitochondria that accumulated PINK1 KD (and so likely accumulated endogenous PINK1, as well) recruited Parkin (Figure 1I). Although Parkin and PINK1 KD colocalize in paraquat-treated cells (average Pearson coefficient = 0.45±0.13), PINK1KD does not colocalize with MTR (average Pearson coefficient = 0.22±0.13; *p*-value = 0.002 for PINK1 KD/Parkin vs. PINK1 KD/MTR) (Figure 1J).

Considered together, these results demonstrate that PINK1 selectively accumulates on dysfunctional mitochondria with low membrane potentials.

### PINK1 Cleavage Is Inhibited by Loss of Membrane Potential, Leading to Its Accumulation on the Outer Mitochondrial Membrane

**Figure 2:**
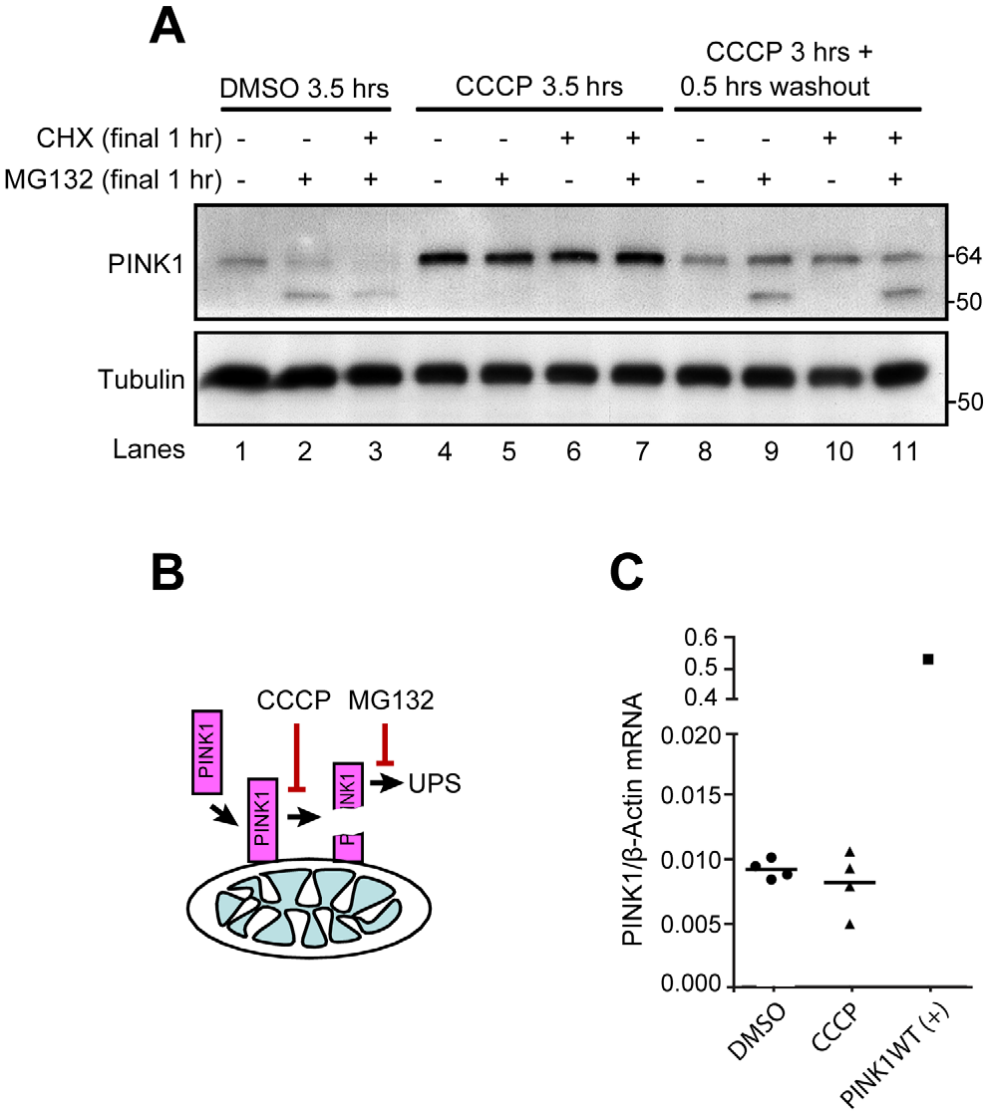
PINK1 accumulates following inhibition of voltage-sensitive cleavage. (A) HeLa cells stably expressing YFP-Parkin were treated with DMSO for 3.5 h, 2 µM CCCP for 3.5 h, or CCCP for 3 h followed by washout of CCCP for 0.5 h in the absence of serum. 50 µM MG132 and/or 100 µM cyclohexamide were added for the last 1 h of treatment. Whole-cell lysates (WCL) run on SDS gels and immunoblotted for endogenous PINK1 and tubulin. (B) Model depicting the two-step processing of PINK1. (C) Quantitative RT-PCR was used to measure relative PINK1 mRNA expression in HeLa cells treated with DMSO or CCCP for 1 h. The graph represents the results from four independent experiments. As a positive control, relative PINK1 mRNA levels were also measured in HeLa cells following exogenous expression of PINK1. PINK1 mRNA expression levels were normalized to the housekeeping gene *β-actin*. WT, wild type.

Regulation of PINK1 expression at the level of transcription or translation would likely not be selective for a subpopulation of mitochondria, and so we assessed whether increased PINK1 expression on damaged mitochondria is achieved by the selective removal of PINK1 from functional mitochondria. Full-length PINK1 (∼63 kDa), which is anchored in the mitochondrial membrane, is proteolytically cleaved into an ∼52-kDa cytosolic fragment that can be degraded by the proteasome [22],[28]–[30]. To test whether PINK1 accumulation following CCCP treatment is due to inhibition of its proteolytic cleavage, we assessed the effect of CCCP washout on PINK1 cleavage. HeLa cells were treated with vehicle (DMSO) or CCCP for 3 h, after which CCCP was either washed out or left in for an additional 30 min. Cycloheximide was either added or left out during the final hour of treatment to control for de novo PINK1 synthesis during the washout period. Following PINK1 accumulation in the continuous presence of CCCP for 3 h, the addition of cycloheximide for 1 h has little effect on the abundance of full-length PINK1, suggesting that once it has accumulated, the ∼63-kDa PINK1 is relatively stable on depolarized mitochondria (Figure 2A, lanes 4 vs. lane 6). However, within 30 min of CCCP washout, ∼63-kDa PINK1 abundance falls dramatically, consistent with its being cleaved and maintained at low abundance on polarized, undamaged mitochondria (Figure 2A, lanes 4–7 vs. lanes 8–11). The residual full-length PINK1 seen following CCCP washout largely represents PINK1 that had accumulated during the 3-h CCCP treatment, as the addition of cycloheximide prior to washout has little effect on its level (Figure 2A, lane 8 vs. lane 10).

To further assess the stability of PINK1 under depolarizing conditions, we performed the same set of experiments in the presence of MG132, an inhibitor of proteasomal degradation. When MG132 is added during the final hour of treatment in HeLa cells treated with vehicle, an ∼52-kDa band appears, consistent with the cleavage product of full-length Parkin described in previous reports [22],[28],[30] (Figure 2A, lane 1 vs. lane 2). The accumulation of this short form of PINK1 following treatment with MG132 suggests that it is unstable under basal conditions, as has been observed previously [22],[30] (Figure 2A, lane 1 vs. lane 2). Interestingly, levels of the short form of PINK1 in the presence of MG132 decrease following depolarization with CCCP for 3.5 hrs, as levels of full-length PINK1 rise (Figure 2A, lane 2 vs. lane 5); but increase following CCCP washout, as levels of full-length PINK1 fall (Figure 2A, lane 2 vs. lane 9). This pattern indicates that the cleavage of full-length PINK1 into the unstable short form is blocked by mitochondrial depolarization and reinstated upon CCCP washout. Taken together, these results support a two-step model for the processing of PINK1: first, full-length PINK1 is cleaved into the ∼52-kDa short form in a voltage-dependent, proteasome-independent manner, and second, the short form of PINK1 is rapidly degraded by the proteasome (Figure 2B). The voltage-dependent processing of PINK1 maintains low levels of PINK1 on healthy polarized mitochondria, but allows for the rapid accumulation of PINK1 on depolarized mitochondria.

Although these experiments suggest that the increased expression of PINK1 is due at least in part to inhibition of PINK1 cleavage, it is possible that increased transcription of PINK1 following depolarization might also be contributing to the increase in PINK1 abundance. To assess whether PINK1 transcription is also regulated by membrane potential, we performed quantitative RT-PCR (qRT-PCR) of PINK1 levels in HeLa cells treated with DMSO or CCCP for 1 h. We found that whereas exogenous expression of PINK1 causes a significant increase in PINK1 transcription relative to untransfected HeLa cells, PINK1 transcription does not significantly increase following depolarization with CCCP (p = 0.4499). These data confirm that the increase in PINK1 expression following depolarization is not driven by an increase PINK1 transcription (Figure 2C).

Finally, to test the localization of accumulated PINK1 on depolarized mitochondria, we performed a protease protection assay, using an antibody raised against PINK1's kinase domain. Consistent with results from a recent study of PINK1's topology when it is ectopically expressed [22], we found that the kinase domain of endogenous PINK1 faces the cytosol following depolarization (Figure S1E).

### PINK1 Accumulation on Depolarized Mitochondria Is Independent of the Protease PARL

The protease responsible for PINK1 cleavage in mammalian cells is unknown, but in *Drosophila* cells, the intramembrane serine protease, Rhomboid-7, appears to be required for PINK1 cleavage [31]. To examine whether the mammalian ortholog of Rhomboid-7, PARL, is responsible for PINK1 cleavage in mammalian cells, we tested whether PINK1-V5 accumulates in HeLa cells transfected with PARL shRNA and treated with CCCP. Although endogenous PARL could not be detected in HeLa cells, PARL shRNA inhibited expression of overexpressed PARL (Figure S2A and S2B). Knockdown of PARL did not appreciably change basal levels of endogenous PINK1 or augment the depolarization-induced accumulation of endogenous PINK1 in HeLa cells (Figure S2B). Likewise, PINK1-V5 levels were similar in PARL^−/−^ and PARL^+/+^ MEFs, under basal conditions and following depolarization with CCCP (Figure S2C). Together, these results suggest that PARL is dispensable for PINK1 cleavage.

### PINK1 Accumulation on Depolarized Mitochondria Is Independent of Parkin Expression

Previous studies in *Drosophila* and mammalian cells indicate that PINK1 functions genetically upstream of Parkin [11]–[13],[20],[32], although the molecular mechanism of this genetic interaction remains unexplained. To test whether PINK1 accumulation on mitochondria is upstream of Parkin recruitment to depolarized mitochondria, we assessed the dependence of PINK1 accumulation on Parkin expression. Endogenous PINK1 accumulates similarly in HeLa cells, which display little or no endogenous Parkin expression, and HeLa cells stably expressing YFP-Parkin (Figure S2D). Consistent with these findings, we observed that exogenous PINK1-myc accumulates similarly in immortalized Parkin^−/−^ and Parkin^+/+^ MEFs (Figure S2E). Together, these results show that PINK1 accumulation is upstream of Parkin recruitment to depolarized mitochondria and independent of Parkin expression.

### PINK1 Expression Is Required for Parkin Recruitment to Depolarized Mitochondria and Parkin-Induced Mitophagy

**Figure 3:**
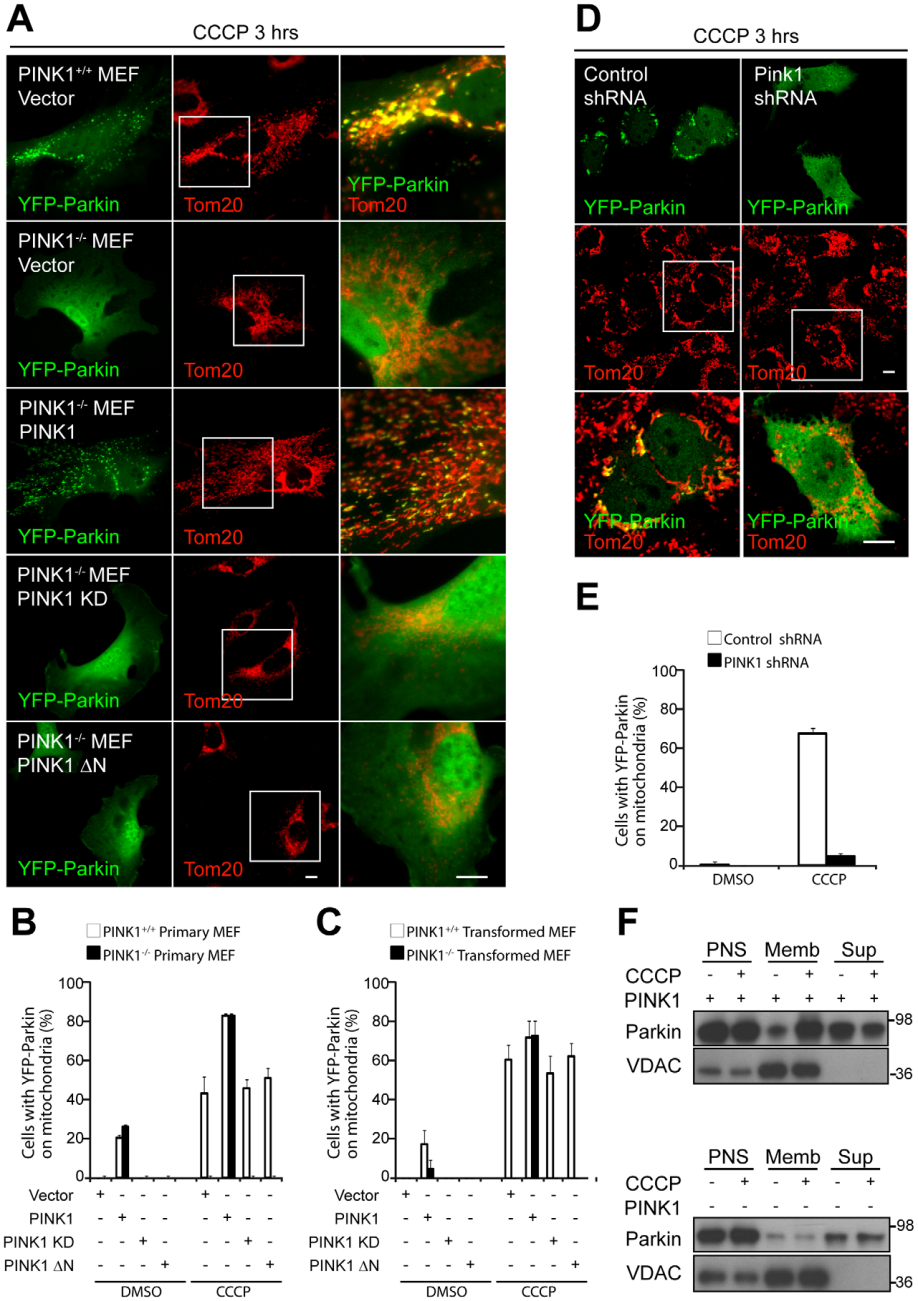
Parkin recruitment to depolarized mitochondria requires PINK1 and its mitochondrial targeting N-terminus. (A) Primary MEFs from PINK1^+/+^ or PINK1^−/−^ mice cotransfected with YFP-Parkin (green) and the indicated construct (vector, PINK1-V5, PINK1 kinase-deficient [KD]-V5, or PINK1 156–581 [ΔN]-V5) in a 1∶4 ratio were treated with DMSO or 20 µM CCCP in serum for 3 h. Mitochondria were immunostained for Tom20 (red). The images in the column on the right, which are merged images of the middle and left-hand columns, are expansions of the boxed regions in the middle column. (B) Colocalization between YFP-Parkin and mitochondria in (A) was scored for ≥100 cells/condition in three or more independent experiments. (C) Transformed MEFs from independently generated PINK1^+/+^ and PINK1^−/−^ mice were transfected and treated as in (A) and were scored as in (B). (D) M17 human neuroblastoma cells stably transduced with control shRNA or PINK1 shRNA were treated with 10 µM CCCP in serum for 3 h and imaged as in (A). (E) Colocalization between YFP-Parkin and mitochondria in (D) was scored as described in (B). (F) Control shRNA and PINK1 shRNA M17 cells transfected and treated as in (D) were fractionated into mitochondria-rich membrane fraction (Memb) and supernatant (Sup). Fractions were run on SDS gels and immunoblotted with anti-Parkin and anti-VDAC antibodies. Loading was adjusted for approximately equal concentrations of YFP-Parkin in the postnuclear supernatants (PNS) between the two cell types. Scale bars in (A and D) represent 10 µm. Error bars in (B, C, and E) indicate standard deviation.

Next, we tested whether Parkin recruitment to depolarized mitochondria is dependent on PINK1 expression. We found that, although YFP-Parkin is recruited to mitochondria in 43.3±8.1% (mean ± standard deviation [SD]) of PINK1^+/+^ primary MEFs after 3-h exposure to 20 µM CCCP, it is not detectably recruited to mitochondria in PINK1^−/−^ MEFs, as assessed by confocal microscopy (Figure 3A and 3B). We also failed to detect YFP-Parkin recruitment at 24 h following CCCP in PINK1^−/−^ MEFs (unpublished data), suggesting that little or no recruitment of YFP-Parkin to depolarized mitochondria occurs in the absence of PINK1. YFP-Parkin recruitment could be reconstituted in PINK1^−/−^ MEFs by expression of wild-type PINK1, but not by PINK1 ΔN lacking its mitochondrial targeting N-terminus (1–155) [22], suggesting that mitochondrial targeting of PINK1 is required for Parkin recruitment to mitochondria (Figure 3A and 3B). A kinase-deficient (KD) version of PINK1 [28] also failed to reconstitute Parkin recruitment to mitochondria (Figure 3A and 3B).

We further tested the dependence of Parkin recruitment on PINK1 in a SV40-transformed MEF cell line, which was derived from an independently generated PINK1^−/−^ mouse [29] (Figure 3C and Figure S3A). Similar to the primary PINK1^−/−^ MEFs, no recruitment is seen in the transformed PINK1^−/−^ cells, whereas Parkin is recruited to mitochondria in 60.7±7.7% of PINK1^+/+^ cells upon CCCP treatment. Likewise, Parkin recruitment in the transformed PINK1^−/−^ cells is reconstituted following exogenous expression of PINK1 (72.8±7.7% vs. 0.0±0.0%, *p*-value <0.001), but not PINK1 ΔN or PINK1 KD.

Finally, we tested the dependence of Parkin recruitment in a human neuroblastoma cell line (M17) [33],[34]. In M17 cells stably transduced with PINK1 shRNA, YFP-Parkin translocated to mitochondria in 4.7±1.2% of CCCP-treated cells, whereas 67.3±3.1% of control shRNA M17 cells displayed mitochondrial YFP-Parkin after treatment with 10 µM CCCP for 3 h (*p*-value <0.001) (Figure 3D and 3E). Vehicle treatment failed to induce YFP-Parkin translocation to mitochondria in both cell lines. The necessity of PINK1 expression for Parkin recruitment to membranes was also examined in the M17 cell line by immunoblotting. In control shRNA cells, YFP-Parkin levels increase in the mitochondria-rich membrane fraction and decrease in the supernatant following treatment with CCCP, consistent with Parkin translocation to mitochondria (Figure 3F, upper panel, and Figure S3B). YFP-Parkin was expressed less in the PINK1 shRNA cells compared to control shRNA cells, possibly because the transfection efficiency is lower in these cells and/or because Parkin is less stable in the absence of PINK1, as has been observed previously [29]. Nonetheless, we failed to see Parkin increase in the membrane fraction either under equal loading conditions or when loading was adjusted so that total Parkin was approximately equal in the two cell populations, further indicating that Parkin is not recruited to uncoupled mitochondria in the absence of PINK1 (Figure 3F, lower panel, and Figure S3B).

**Figure 4:**
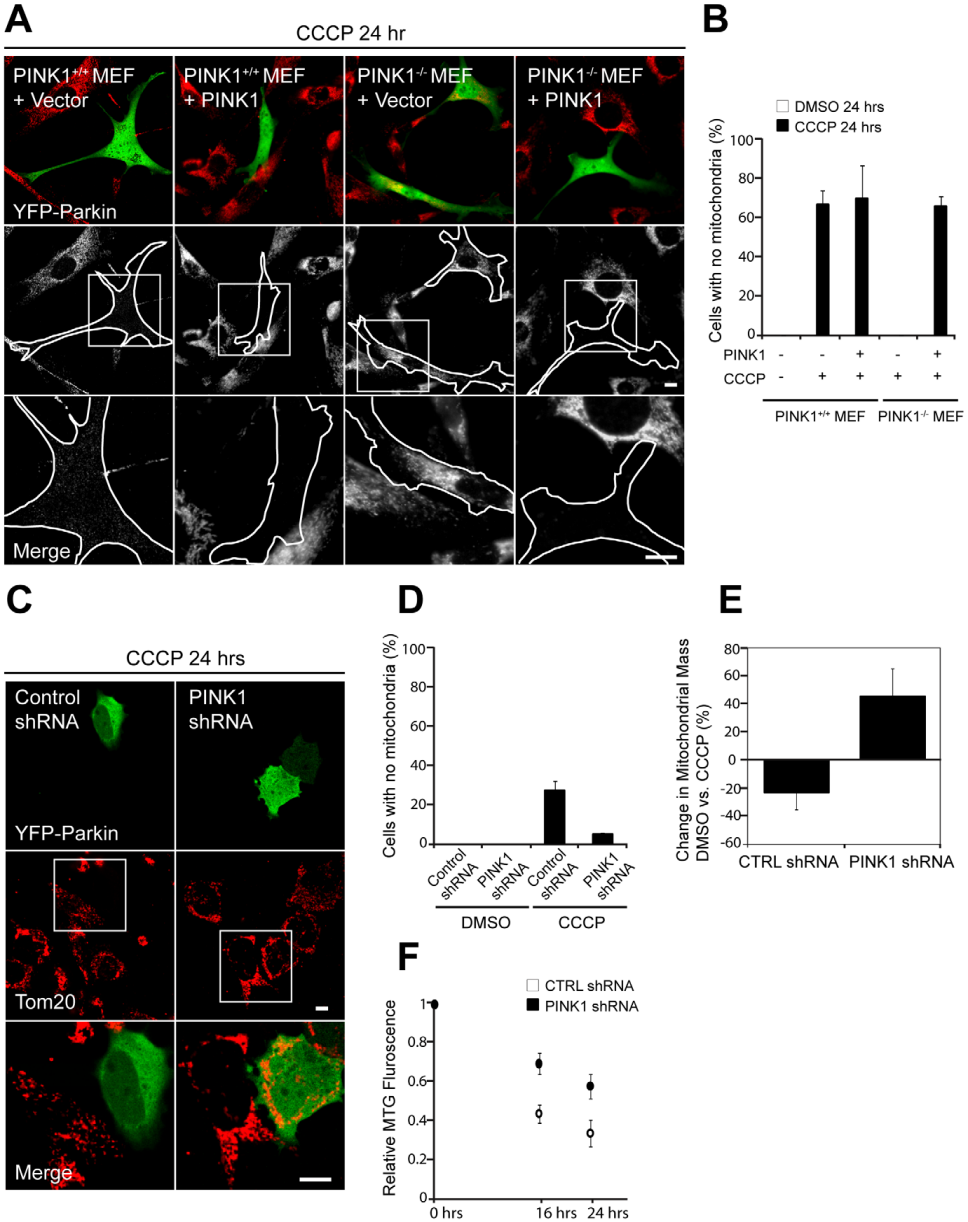
PINK1 is required for Parkin-induced autophagy of depolarized mitochondria. (A) Primary MEFs from PINK1^+/+^ or PINK1^−/−^ mice cotransfected with YFP-Parkin were treated with DMSO or 20 µM CCCP in serum for 24 h. Mitochondria were stained with an anti-Tom20 antibody. (B) Percentage of cells with no detectable mitochondria in (A) was scored for >150 cells/condition in three or more independent experiments. (C) M17 human neuroblastoma cells stably transduced with control shRNA or PINK1 shRNA were treated with 10 µM CCCP for 24 h and stained as in (A). Images in the bottom rows of (A and C) are expansions of the images indicated by the boxes in the middle rows. Scale bars in (A and C) represent 10 µm. (D) Percentage of cells with no mitochondria was scored for (C) as described in (B). (E) M17 cells stably transduced with control shRNA or PINK1 shRNA were treated with DMSO or 10 µM CCCP for 24 h and stained with Mitotracker Green (MTG). MTG, which stains mitochondrial lipid in a membrane potential independent manner, is a sensitive measure of mitochondrial mass. The graph represents change in Mitotracker Green intensity between DMSO- and CCCP-treated samples in three independent experiments. (F) M17 cells stably transduced with control shRNA or PINK1 shRNA were pulsed with Mitotracker Green in the presence of CCCP. Loss of MTG intensity was measured at 0 h, 16 h, and 24 h with a plate reader. The graph shows data from three biological replicates and is representative of three independent experiments. The error bars in (B, D, and E) indicate the standard deviation.

We previously reported that ectopic Parkin can induce the autophagy of depolarized mitochondria [21]. To test whether PINK1 is necessary for Parkin-induced mitophagy, we treated primary PINK1^−/−^ and PINK1^+/+^ MEFs transiently expressing YFP-Parkin with 20 µM CCCP for 24 h (Figure 4A and 4B). Whereas no mitochondria can be detected in 66.1±16.8% of PINK1^+/+^ MEFs, all PINK^−/−^ MEFs retain their mitochondria. Parkin-dependent mitophagy is reconstituted by exogenous PINK1 expression in the PINK^−/−^ MEFs, with 65.5±5.0% of reconstituted PINK^−/−^ cells displaying undetectable mitochondria following CCCP treatment.

We found that Parkin-induced mitophagy is also dependent on PINK1 expression in the M17 human neuroblastoma cell line. Whereas in 27.1±8.6% of control shRNA M17 cells displayed complete loss of mitochondria after 24 h, less than 5% of cells lost mitochondria in the PINK1 shRNA cells (Figure 4C and 4D). These results suggest that PINK1 is necessary for the mitophagy of depolarized mitochondria following overexpression of Parkin.

To test whether PINK1 expression affects mitochondrial turnover in the presence of endogenous levels of Parkin, we treated the control shRNA and PINK1 shRNA M17 cells (which express moderate levels of Parkin) with DMSO or CCCP for 24 h and measured their relative mitochondrial mass by Mitotracker Green (MTG) staining and flow cytometry. MTG, a sensitive measure of mitochondrial mass, stains mitochondrial lipid in a membrane potential–independent manner and has been used to measure mitochondrial mass of depolarized mitochondria previously [35],[36]. Control shRNA M17 cells exhibit a decrease in mitochondrial mass (CCCP vs. DMSO, −22.4±12.6%) following CCCP treatment, whereas PINK1 shRNA M17 cells exhibit an increase in mitochondrial mass (CCCP vs. DMSO, 43.5±20.0%) following depolarization (*p*-value = 0.008 for change in mitochondrial mass control shRNA vs. PINK shRNA) (Figure 4E). These results are consistent with endogenous PINK1 promoting mitochondrial degradation in the context of continued (or increased) mitochondrial biogenesis [37],[38]. To more directly assay mitochondrial turnover in control and PINK1 shRNA M17 cells, we pulsed the cells with MTG and tracked loss of MTG intensity at 0, 16, and 24 h in the presence of CCCP. Consistent with the hypothesis that endogenous PINK1 promotes the degradation of depolarized mitochondria, MTG intensity decreases more slowly in PINK1 shRNA cells when compared with control shRNA cells, treated with CCCP (0.58±0.07 vs. 0.33±0.07 relative MTG intensity at 24 h) (Figure 4F). These data suggest that PINK1 promotes mitophagy in the context of endogenous levels of Parkin. Additionally, these results suggest that the selective turnover of dysfunctional mitochondria may be balanced by the biogenesis of new mitochondria, allowing exchange of damaged, dysfunctional mitochondria for healthy, functional mitochondria.

Consistent with genetic studies in *Drosophila*, these findings show that Parkin translocation to depolarized mitochondria and Parkin-induced mitophagy are downstream of PINK1 expression, whereas PINK1 accumulation in response to depolarization is upstream of Parkin recruitment.

### Expression of PINK1 on the Outer Mitochondrial Membrane Is Sufficient for Parkin Recruitment and Mitophagy

**Figure 5:**
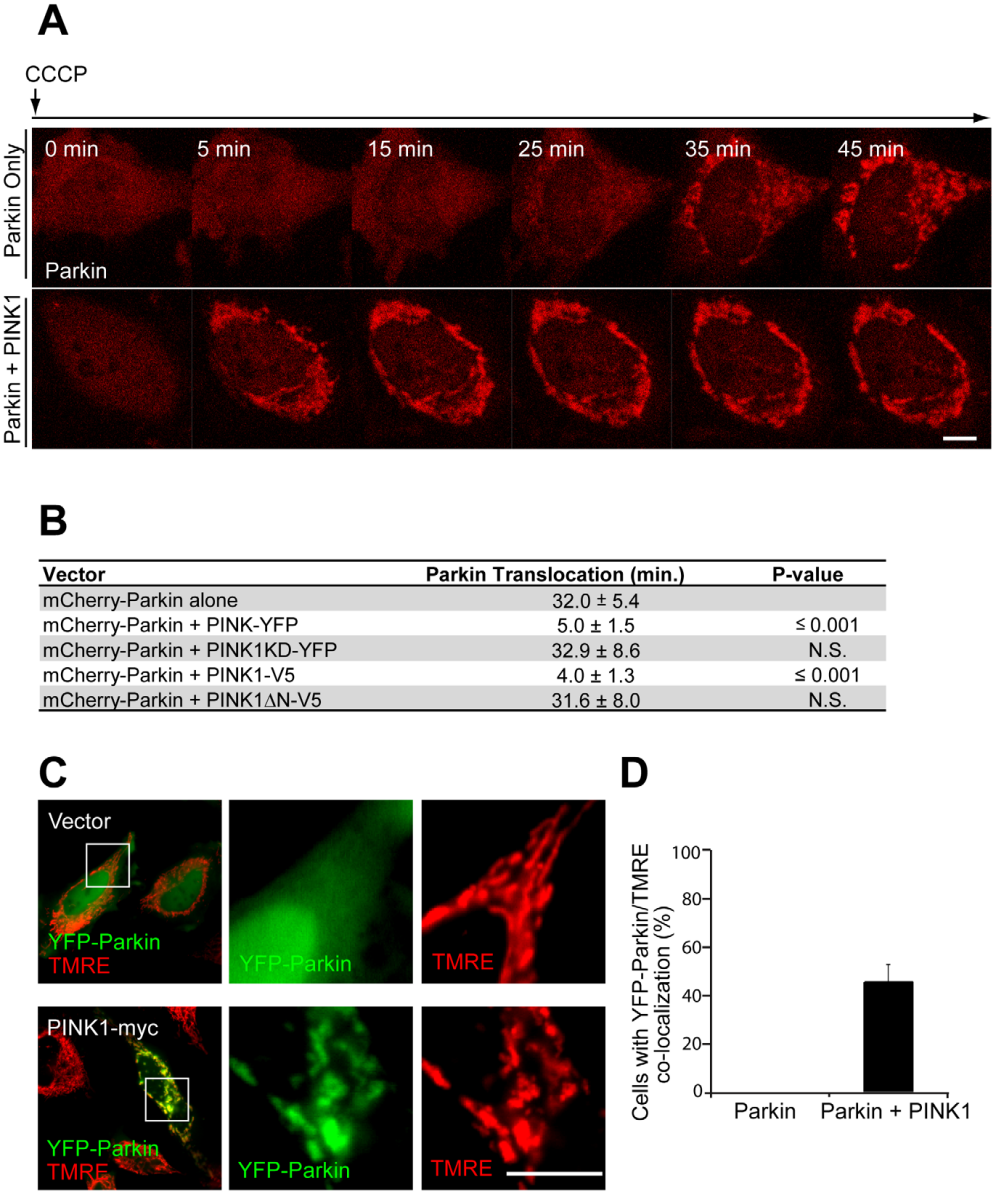
Kinetics of Parkin recruitment are modulated by PINK1 expression. (A) HeLa cells transfected with mCherry-Parkin (red) alone or mCherry-Parkin (red) and PINK1-YFP in a 1∶1 ratio were imaged live following the addition of 10 µM CCCP in serum at time point 0 min. (B) HeLa cells transfected with mCherry-Parkin and the indicated construct in a 1∶1 ratio were treated as in (A) and imaged live (one frame/minute) following the addition of CCCP. Time to the beginning of Parkin translocation was defined as the first appearance of puncta in two or more quadrants of the cell for two or more consecutive images for six or more cells in a minimum of three independent experiments. N.S., nonsignificant. (C) Live confocal image of HeLa cells transfected with YFP-Parkin (green) or YFP-Parkin (green) and PINK1-myc (in a 1∶4 ratio). Cells were loaded with TMRE (red) to stain polarized mitochondria. Cells were not treated with CCCP. Scale bar in last image represents 10 µm. Images in the middle and right-hand panels are expansions of the boxed regions in the panels on the left. (D) Cells treated as described in (C) were scored for colocalization between YFP-Parkin and TMRE. ≥50 cells/experiment were scored in three or more independent experiments.

The expression of mitochondrial PINK1 is necessary for recruitment of Parkin to mitochondria. Next, we tested whether PINK1 overexpression is sufficient for Parkin recruitment to mitochondria. Using live-cell imaging, we found that moderate overexpression of PINK1 dramatically accelerates the kinetics of Parkin recruitment following depolarization with CCCP (time to translocation 5.0±1.5 min vs. 32.0±5.4 min, *p*-value <0.001) (Figure 5A and 5B; Video S2 vs. S3). Consistent with necessity of PINK1's mitochondrial localization and kinase activity, exogenous expression of PINK1 KD or PINK1 ΔN fails to accelerate the kinetics of Parkin recruitment (Figure 5B). In cells with high expression of PINK1, Parkin is recruited to mitochondria even in the absence of CCCP (Figure 5C and 5D), as has been reported previously [27]. YFP-Parkin colocalizes with the potentiometric mitochondrial dye TMRE in 45.3±7.6% of cells coexpressing YFP-Parkin and PINK1 vs. 0±0% of cells expressing Parkin alone (*p*-value <0.001) (Figure 5C and 5D). Together, these results demonstrate that the kinetics of Parkin recruitment is exquisitely sensitive to PINK1 levels in the cell. In addition, they indicate that increased PINK1 expression is sufficient for Parkin recruitment independent of membrane potential.

**Figure 6:**
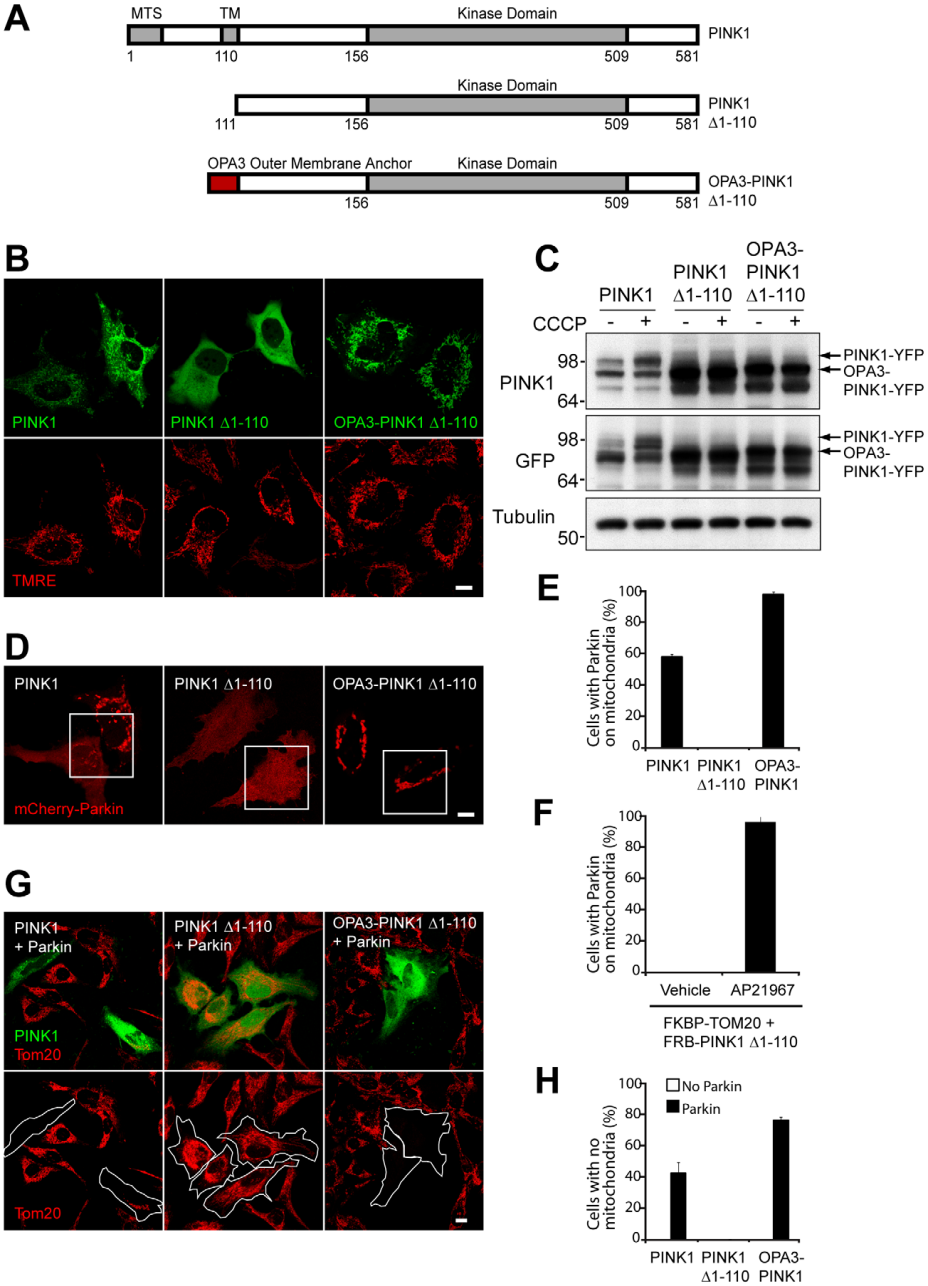
Stable expression of PINK1 on the outer mitochondrial membrane is sufficient for Parkin recruitment. (A) Schematic diagram depicting the construction of PINK1-YFP (green), PINK1 (111–581)-YFP (green), and OPA3-PINK1 (111–581)-YFP (green). (B) Confocal images depicting the localization of PINK1-YFP, PINK1 (111–581)-YFP, and OPA3-PINK1 (111–581)-YFP in HeLa cells. Mitochondria are stained with the potentiometric dye TMRE (red). (C) HeLa cells were transfected with PINK1-YFP, PINK1 (111–581)-YFP, or Opa3-PINK1 (111–581)-YFP and treated with DMSO or 2 µM CCCP in serum-free medium for 3 h. Whole-cell lysates (WCL) were run on SDS gels and immunoblotted for PINK1, GFP, and tubulin. (D) Confocal images of HeLa cells cotransfected with mCherry-Parkin (red) and PINK1-YFP (green), PINK1 (111–581)-YFP (green), or OPA3-PINK1 (111–581)-YFP (green). Cells were not treated with CCCP. (E) HeLa cells in (D) were scored for mCherry-Parkin forming puncta characteristic of mitochondria in ≥150 cells in three or more independent experiments. Cells were not treated with CCCP. (F) HeLa cells were transfected with FRB-PINK1 (111–581)-YFP, which is in the cytosol, TOM20(1–33)-FKBP, which is on mitochondria, and mCherry-Parkin. In the presence of the rapamycin analog, AP21967, the FRB and FKBP domains of the respective fusion proteins (PINK1 (111–581) and TOM20's outer mitochondrial membrane anchor) heterodimerize, if they have access to the same compartment (e.g., the cytosol). Cells treated with vehicle or 250 nM AP21967 for 8 h were scored for mCherry-Parkin in puncta characteristic of mitochondria in ≥150 cells in three or more independent experiments. (G) Confocal images of HeLa cells transfected with PINK1-YFP (green), PINK1 (111–581)-YFP (green), or OPA3-PINK1 (111–581)-YFP (green) with or without ECFP-Parkin and cultured for 96 h in the absence of CCCP. Cells were immunostained for Tom20 (red). To aid in visualizing cells that lack mitochondria, some individual cells have been outlined. (H) Cells treated as in (G) were scored for the absence of detectable mitochondria in ≥150 cells in three or more independent experiments. Scale bars in all images represent 10 µm.

To test whether stable expression of PINK1 on the mitochondria is sufficient for Parkin recruitment, we constructed a fusion protein that would be predicted to lack PINK1's proteolytic cleavage site and therefore exhibit greater stability on mitochondria. Based on the ∼11-kDa difference between the full-length form and the cleaved form, the cleavage site likely lies before residue 110 (residues 1–110 have a predicted molecular weight of 11.54 kDa), and so we replaced residues 1–110 of PINK1 with the outer mitochondrial membrane anchor from OPA3 (1–30) (Figure 6A). We found that removing the first 110 amino acids of PINK1 prevents targeting of PINK1 to mitochondria, consistent with previous reports [39] (Figure 6B, middle panel); whereas the fusion of OPA3 (1–30) to PINK1 Δ1-110 restores mitochondrial targeting, likely to the outer mitochondrial membrane (Figure 6B, right panel). As predicted by the proteolytic cleavage results (Figure 2A), OPA3-PINK1 Δ1-110-YFP exhibits increased stability compared to PINK1-YFP (Figure 6C). In addition, OPA3-PINK1 Δ1-110-YFP levels do not respond to mitochondrial depolarization with CCCP, indicating that stabilization of PINK1 by depolarization depends on its first 110 amino acids. When coexpressed with mCherry-Parkin, PINK1-YFP recruits mCherry-Parkin to mitochondria in 57.9±1.8% of cells in the absence of CCCP; whereas PINK1 Δ1-110-YFP, which is not expressed on mitochondria, fails to recruit mCherry-Parkin in the absence of CCCP. However, OPA3-PINK1 Δ1-110-YFP, which does not display voltage dependent proteolysis, recruits mCherry-Parkin to mitochondria in 98±1.8% of cells in the absence of CCCP (Figure 6D and 6E). Together, these data demonstrate that stable expression of PINK1 on mitochondria is sufficient for Parkin recruitment to mitochondria, regardless of membrane potential.

To verify that increased expression of PINK1 on the outer mitochondrial membrane is sufficient to induce Parkin recruitment, we used a regulated heterodimerization system [40], in which the modified FRB domain was fused to PINK1 Δ1-110-YFP and the FKBP domain was fused to the outer mitochondrial membrane anchor of TOM20 (residues 1 through 33) (Figure S4A). In the presence of the rapamycin derivative AP21967, the FRB domain and the FKBP domain heterodimerize, but only if they are in the same compartment. Thus, FRB-PINK1Δ1-110-YFP should be recruited from the cytosol to mitochondria if the FKBP domain of TOM20-FKBP faces the cytosol but not if it faces the inter membrane space or the matrix. We found that FRB-PINK1Δ1-110-YFP is in the cytosol in the absence of AP21967, but is quickly recruited to the outer mitochondrial membrane following the addition of AP21967 (Figure S4B and S4C). To assess whether PINK1 expression on the outer mitochondrial membrane is sufficient to recruit Parkin, we cotransfected mCherry-Parkin, FRB-PINK1Δ1-110-YFP, and TOM20-FKBP. In the absence of AP21967, mCherry-Parkin is in the cytosol, but following incubation with AP21967 mCherry-Parkin is recruited to mitochondria in 96.7±4.1% of cells, in the absence of CCCP (Figure 6F and Figure S4D). Thus, increased expression of PINK1 on the outer mitochondrial membrane is sufficient to recruit Parkin to mitochondria.

Next, we tested whether Parkin recruitment following increased PINK1 expression on the mitochondria is sufficient to induce mitophagy in the absence of depolarization with CCCP. Cotransfection of PINK1 and Parkin results in a substantial proportion of cells (42.1±7.3%) with no mitochondria after 96 h. By contrast, cotransfection of cytosolic PINK1 Δ1-110-YFP with Parkin produces no cells lacking mitochondria after 96 h. Fusing the outer membrane anchor of OPA3 to PINK1 Δ1-110-YFP, which results in stable expression of PINK1 on mitochondria, restores the ability of PINK1 and Parkin to induce mitophagy ,with 76.4±2.2% of cells lacking mitochondria at 96 h (Figure 6G and 6H). These data demonstrate that stable expression of PINK1 on the outer mitochondrial membrane is sufficient to induce mitophagy in the presence of Parkin, irrespective of membrane potential.

### PINK1 Accumulation following Depolarization Is Necessary for Parkin Recruitment to Mitochondria

**Figure 7:**
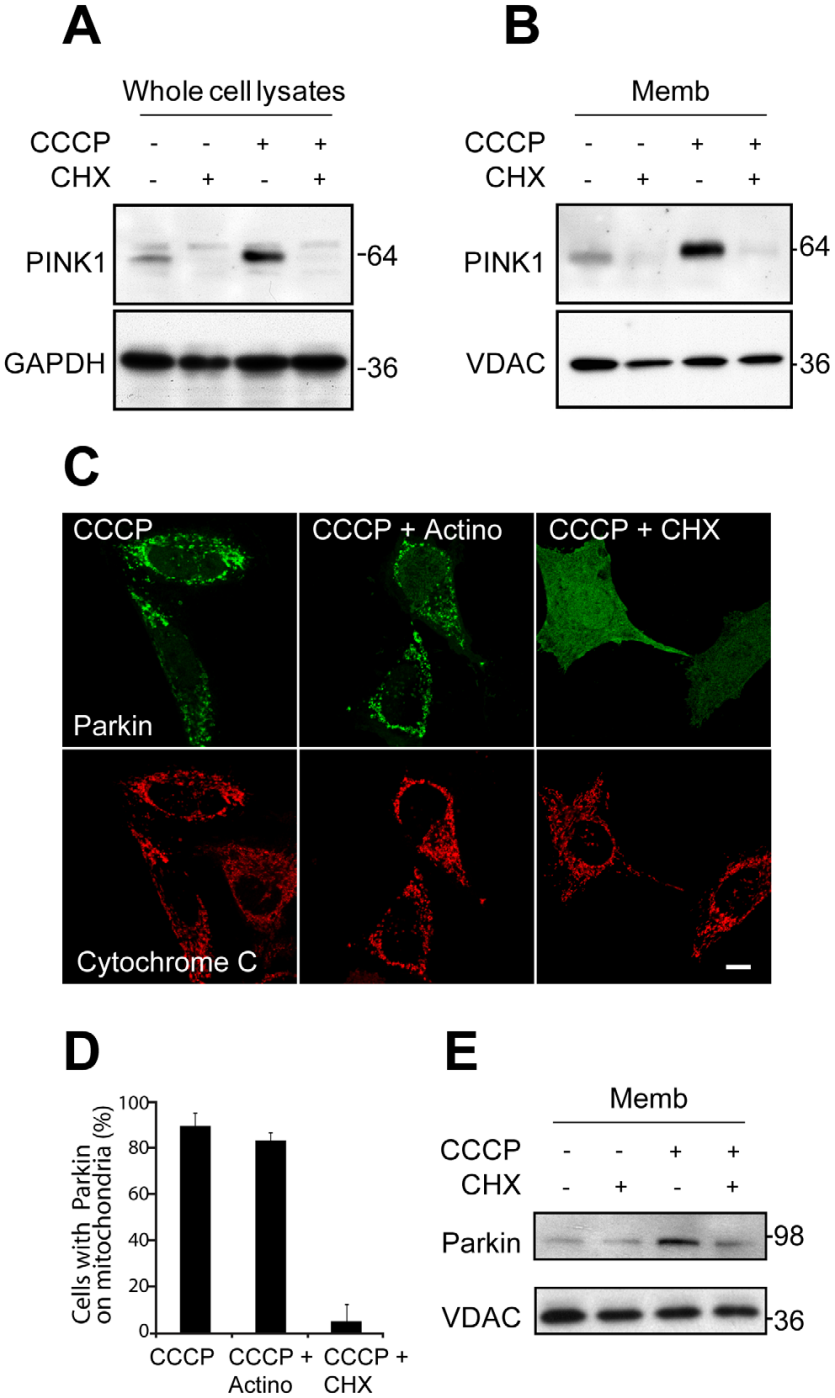
PINK1 accumulation following depolarization with CCCP may be required for Parkin recruitment. (A) HeLa cells stably expressing YFP-Parkin were treated with 2 µM CCCP 1 h alone or CCCP 1 h + 2 µM CHX (30-min pretreatment and 1-h treatment) in the absence of serum. Whole-cell lysates were run on SDS gels and immunoblotted for endogenous PINK1 and the loading control GAPDH. (B) Cells treated as in (A) were fractionated. The mitochondria-enriched membrane fraction (Memb) was run on SDS gels and immunoblotted for endogenous PINK1 and VDAC. (C) HeLa cells were transfected with YFP-Parkin (green) and treated with 10 µM CCCP 1 h alone, CCCP + 10 µM of actinomycin (30-min pretreatment and 1-h treatment), or CCCP 1 h + 100 µM CHX (30-min pretreatment and 1-h treatment) in the presence of serum and immunostained for Tom20 (red). (D) Colocalization between YFP-Parkin and mitochondria in (C) was scored for ≥150 cells/condition in three or more independent experiments. (E) HeLa cells stably expressing YFP-Parkin were treated as in (A) and fractionated. The mitochondria-rich fraction was run on an SDS gel and immunostained for Parkin. Scale bars in all images represent 10 µm.

To test whether accumulation of endogenous PINK1 following depolarization is necessary for Parkin recruitment, we treated HeLa cells with CCCP alone (for 60 min) or with CCCP plus cycloheximide, a general inhibitor of protein synthesis (cycloheximide was added 30 min before CCCP and maintained throughout the 60-min CCCP treatment). Treatment of HeLa cells for 90 min with cycloheximide blocked the depolarization-induced accumulation of endogenous PINK1 in whole-cell lysates as well as in the mitochondria-rich membrane fraction (Figure 7A and 7B). A 90-min treatment with cycloheximide, likewise, blocked Parkin recruitment to depolarized mitochondria by confocal microscopy (96.0±3.5% vs. 11.3±4.2%) (Figure 7C and 7D). By contrast, 90-min treatment with actinomycin D, an inhibitor of transcription, had a modest effect on Parkin recruitment to uncoupled mitochondria by confocal microscopy (Figure 7C and 7D), suggesting that new transcription of PINK1 is not required for Parkin recruitment. This is consistent with the absence of PINK1 mRNA up-regulation following uncoupling (Figure 2C). Cycloheximide likewise blocked YFP-Parkin accumulation in the mitochondria-enriched heavy membrane fraction by immunoblotting (Figure 7E). Although these findings do not prove new PINK1 synthesis is required for Parkin recruitment (cycloheximide blocks de novo synthesis of all proteins and thus may inhibit Parkin recruitment independently of PINK1 accumulation), they suggest that PINK1 accumulation and Parkin recruitment may be casually related.

### Threonines 175 and 217 in Parkin May Not Be Involved in Parkin Recruitment to Mitochondria

It has been proposed that PINK1 may induce mitochondrial recruitment of Parkin through phosphorylation of threonines 175 and 217 in a highly conserved region/domain of Parkin, which has been recently named RING0 (Figure S5A) [27],[41]. We found that although mutation of T175 and T217 to alanine blocked recruitment of Parkin to mitochondria, as was reported previously, the phosphomimetic mutants T175E, T217E, and T175, 217E do not translocate to mitochondria spontaneously. In addition, these phosphomimetic mutants appear to inhibit CCCP-induced recruitment of Parkin. Although these findings do not rule out the possibility that phosphorylation of these sites by PINK1 or another kinase induces Parkin recruitment, they suggest that these threonines are more likely to play an important structural role (Figure S5B and S5C).

### Patient Mutations in PINK1 and Parkin Disrupt PINK1/Parkin Pathway at Distinct Steps

**Figure 8:**
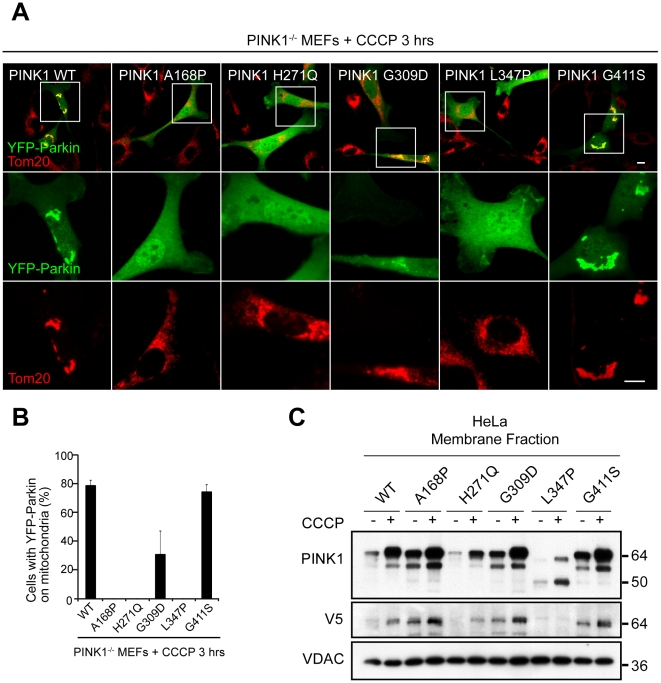
Disease-causing PINK1 mutants fail to reconstitute Parkin recruitment to depolarized mitochondria. (A) Primary MEFs from PINK1^−/−^ mice cotransfected with YFP-Parkin (green) and indicated V5-tagged PINK1 constructs in a 1∶4 ratio were treated with DMSO or 20 µM CCCP in serum for 3 h. Mitochondria were stained with an anti-Tom20 antibody (red). Scale bar in images represents 10 µm. Images in the middle and bottom rows are expansions of the images indicated by the boxes in the top row. (B) Colocalization between YFP-Parkin and mitochondria in (A) was scored for >150 cells/condition in three or more independent experiments. Error bars indicate standard deviation. (C) HeLa cells stably expressing YFP-Parkin were transfected with the indicated V5-tagged constructs, treated with DMSO or 2 µM CCCP for 3 h in serum-free medium, and fractionated. The mitochondria-rich membrane fraction was run on an SDS gel and immunoblotted for PINK1, the V5 tag, and the mitochondrial protein VDAC.

We assessed the ability of disease-causing mutations in PINK1 to reconstitute YFP-Parkin recruitment to mitochondria in PINK1^−/−^ primary MEFs. Following exogenous PINK1 WT expression in PINK1^−/−^ MEFs, YFP-Parkin was recruited to mitochondria in 78.6±3.9% of cells after 20 µM CCCP treatment for 3 h (Figure 8A and 8B). We found that the L347P patient mutant of PINK1 is unstable (Figure 8C and Figure S6A), as was reported previously [28], and that L347P failed to reconstitute YFP-Parkin recruitment to depolarized mitochondria (Figure 8A and 8B). Of the patient mutations that exhibited stable expression, A168P and H271Q also failed to reconstitute YFP-Parkin recruitment at 3 h, whereas G309D only partially reconstituted YFP-Parkin recruitment (30.7±16.7%) (Figure 6A and 6B). The polymorphism G411S, which to date has only been found in cases heterozygous for the mutation [42], reconstituted YFP-Parkin recruitment to a similar extent as wild-type PINK1 (74.2±5.4%), suggesting that PINK1 containing this polymorphism may be functional in the PINK1/Parkin pathway (Figure 8A and 8B). This is consistent with the idea that G411S may represent a natural variant and may not be a true disease-causing mutation. Protein levels of all PINK1 mutants accumulated upon exposure of cells to CCCP (Figure 8C and Figure S6A).

**Figure 9:**
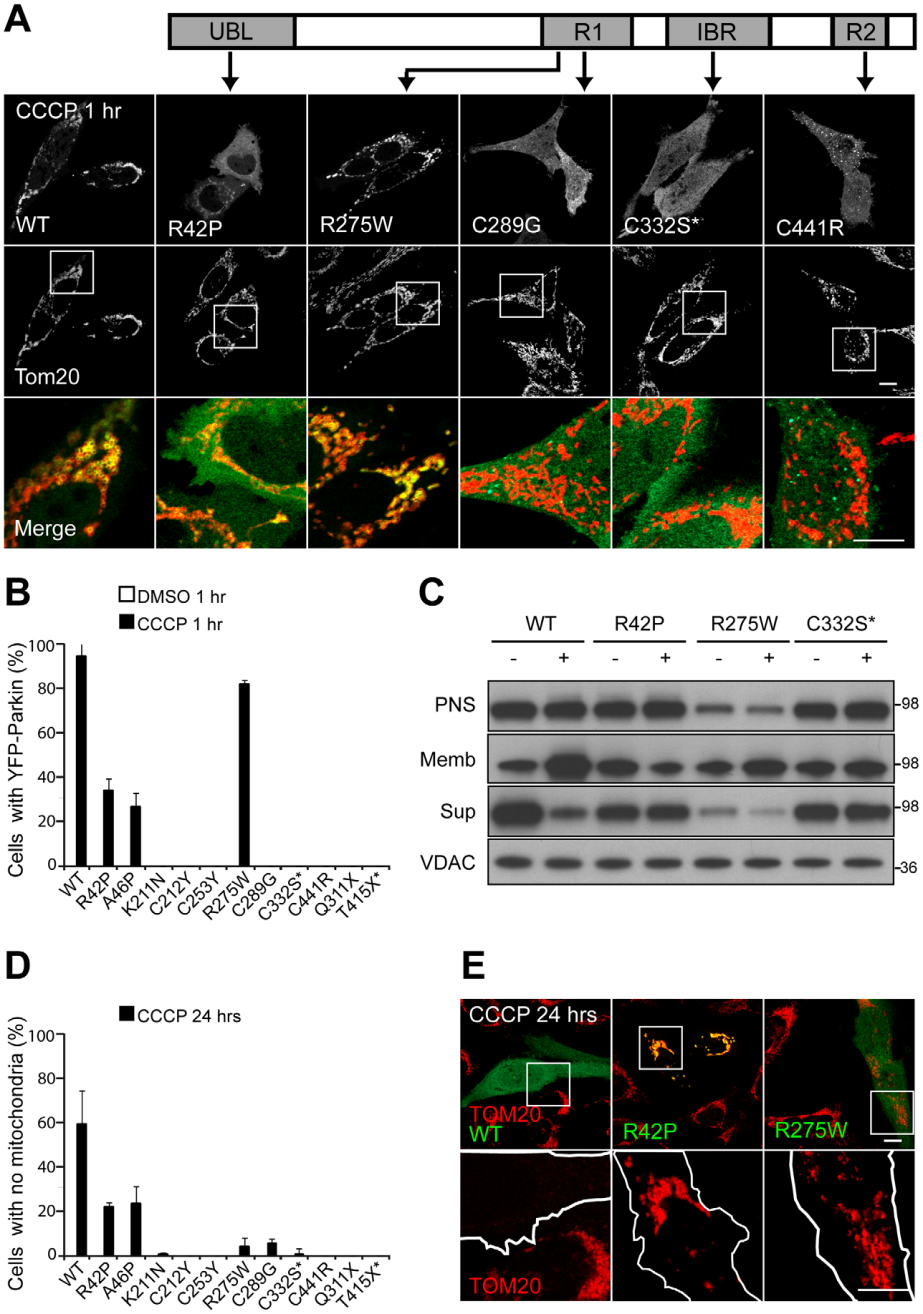
Disease-causing mutations in Parkin disrupt Parkin recruitment to mitochondria and/or Parkin-induced mitophagy. (A) HeLa cells were transfected with YFP-Parkin (white and green) containing indicated mutations and treated with CCCP for 1 h. Mitochondria labeled with an anti-Tom20 antibody (red). Images in the bottom row are expansions of the regions indicated by the boxes in the middle row. WT, wild type. (B) Colocalization between YFP-Parkin and mitochondria in (A) scored for ≥150 cells/condition in three or more independent experiments. (C) HeLa cells transfected and treated as in (A) and fractionated into postnuclear supernatant (PNS), mitochondria-rich heavy membrane fraction (HMF), and supernatant (Sup). Fractions run on SDS gels and immunoblotted for Parkin and VDAC. (D and E) HeLa cells transfected as in (A) and treated with CCCP or DMSO for 24 h. (D) Number of HeLa cells with no mitochondria scored for ≥150 cells/condition in three or more independent experiments. (E) Images of WT, R42P, and R275W Parkin (green) stained as in (A). An asterisk (*) indicates engineered mutation; all others have been linked to Parkinson disease. Scale bars in all images represent 10 µm. Error bars in (B and D) indicate standard deviation.

Next, we tested patient mutations in Parkin to see if they would affect Parkin recruitment to mitochondria and/or Parkin-induced mitophagy. Parkin has an N-terminal ubiquitin-like domain (UBL) and a C-terminal RING-between-RING (RBR) superdomain, which consists of three atypical RING domains (Figure 9A). The fold of the N-terminal RING1 most closely resembles that of traditional RING domains, such as that of c-CBL, whereas the In-Between-RING (IBR) and the C-terminal RING2 likely have unique folds [43]–[45]. The RBR domain is responsible for Parkin's ubiquitin ligase activity, whereas its UBL domain is thought to mediate interactions between Parkin and proteins with ubiquitin-binding domains (UBDs) [46].

As was reported previously, wild-type YFP-Parkin is recruited to mitochondria in the majority of HeLa cells (94.7±5.8%) by confocal microscopy, following treatment with 10 µM CCCP for 1 h (Figure 9A and 9B). Pathogenic mutations in the UBL domain (R42P and R46P), deletion of the UBL, or mutation of a key residue (I44A) in the interaction of UBLs with UBDs [47], all cause a moderate deficit in Parkin recruitment to depolarized mitochondria (34±5.3% and 26.5±6.6% for R42P and R46P, respectively) (Figure 9A and 9B and Figure S7A–S7E). Mutations in conserved cysteines of the RING domains (the patient mutations C253Y, C289G, and C441R and the engineered mutation C332S) completely disrupt recruitment at 1 h of CCCP treatment, as do mutations (patient mutation Q311X and engineered mutation T415X) that result in loss of RING2 (Figure 9A and 9B). Mutations K211N and C212Y, which lie within a highly conserved region of Parkin that is likely a novel RING-like domain [41] (Figure S5A), similarly blocked the recruitment of Parkin to mitochondria (Figure 9B), consistent with the importance of this region for Parkin's activity. Mitochondrial recruitment was seen for several of the conserved cysteine RING mutants (C289G, C332S, and C441R) after 24 h of CCCP exposure, suggesting that recruitment is not completely disrupted with these mutations (Figure S8A and S8B). Interestingly, the R275W mutation in RING1 exhibited only a mild deficit in recruitment (81.7±2.1%) (Figure 9A and 9B). The recruitment of YFP-Parkin R275W was verified in a live-cell imaging experiment (Figure S8C). Although under control conditions some mutants formed visible aggregates (Figure S8D), no mutant, including R275W, colocalized with mitochondria (Figure 9B and Figure S8E).

Next, we assessed recruitment of Parkin mutants to depolarized mitochondria by immunoblotting. As with our previous results [21], we found some background YFP-Parkin signal in the membrane fraction under control conditions. Following treatment with CCCP for 1 h, levels of wild-type Parkin increase in the mitochondria-rich membrane fraction and decrease in the supernatant (Figure 9C). Although expression of Parkin R275W was moderately less than wild type, it also increases localization in the membrane fraction and decreases in the supernatant upon CCCP treatment, consistent with the mitochondrial translocation seen for this mutation by confocal microscopy (Figure 9C). No membrane translocation was detectable by Western blotting for either R42P or C332S, however, suggesting that translocation of these mutants is substantially lower than wild type and consistent with the deficit in mitochondrial translocation seen by confocal microscopy (Figure 9C).

We assessed the ability of Parkin mutants to induce mitophagy. As we found previously [21], expression of wild-type Parkin in HeLa cells that do not detectably express endogenous Parkin completely eliminates mitochondria in greater than half of the cells (59.0±15.1%) following treatment with CCCP for 24 h (Figure 9D and 9E). Mutations in the UBL of Parkin exhibit a moderate loss in mitophagy activity (22.0±2.0% and 23.1±8.4% of cells exhibited no mitochondria for R42P and R46P, respectively); whereas mutations in the conserved cysteines of the RBR or truncations that resulted in loss of RING2 exhibit a severe mitophagy deficit (0±0% to 5.3±2.3%, depending on the mutation) (Figure 9D and 9E). In addition, patient mutations R211N and C212Y caused a similar deficit in mitophagy, supporting the notion that this may be an atypical RING domain similar to RING1, IBR, and RING2 (Figure 9D). Interestingly, the R275W mutation in RING1 also exhibited a severe deficit in mitophagy (4.0±4%) (Figure 9D and 9E) even though it appears to largely retain its ability to translocate to uncoupled mitochondria (Figure 9A and 9B). This pattern of findings suggests that recruitment of Parkin to mitochondria and its induction of mitophagy are dissociable events.

## Discussion

We recently reported that the Parkinson disease-linked E3 ubiquitin ligase, Parkin, is selectively recruited to dysfunctional mitochondria with low membrane potential to promote their autophagic degradation, suggesting that a deficiency of mitochondrial quality control may underlie the observed mitochondrial dysfunction in Parkin knockout *Drosophila* and mice [11]–[15],[17],[21]. How Parkin is able to distinguish damaged, depolarized mitochondria from healthy, polarized mitochondria, however, was unknown.

Here, we show that PINK1 selectively accumulates on depolarized mitochondria that have sustained damage. This selective accumulation is achieved by a novel mechanism, in which PINK1 is constitutively synthesized and imported into all mitochondria, but cleaved from healthy mitochondria by voltage-sensitive proteolysis (Figure S9). On damaged mitochondria that have lost their membrane potential, however, PINK1 cleavage is inhibited, leading to high PINK1 expression on the dysfunctional mitochondria. Expression of mitochondrial PINK1 is required for the recruitment of Parkin to the dysfunctional mitochondria and for their selective elimination by Parkin. In addition, increased expression of PINK1 on the outer mitochondrial membrane is sufficient for Parkin recruitment and Parkin-induced mitophagy, suggesting that loss of membrane potential activates Parkin recruitment primarily through the up-regulation of mitochondrial PINK1.

This model offers a parsimonious explanation for several observations that have been made previously. Full-length mitochondrial PINK1 (∼63 kDa) is cleaved into a short ∼52-kDa form, but the short, primarily cytosolic form is unstable, raising the questions: why is PINK1 found both on the mitochondria and in the cytosol, and which form of PINK1 is active in the PINK1/Parkin pathway [22],[28]–[30]. Our results suggest that full-length mitochondrial PINK1 is the active form in the PINK1/Parkin pathway, and that cleavage of PINK1 into an unstable cytosolic form maintains low levels of PINK1 on healthy mitochondria in order to suppress the PINK1/Parkin pathway in the absence of mitochondrial damage. Additionally, this model provides an explanation for the observation that the uncoupler valinomycin (which can inhibit the TIM22/23 mitochondrial import pathway) blocks PINK1 processing but fails to block PINK1 import [30]. Our model suggests that membrane potential is not required for PINK1 import but is required to selectively maintain low PINK1 expression on healthy mitochondria. This mechanism couples the collapse of mitochondrial voltage potential following mitochondrial damage to selective PINK1 accumulation on damaged mitochondria.

At present, it is unclear which protease(s) mediate the cleavage of PINK1 in mammalian cells. Although the intramembrane serine protease Rhomboid-7 appears to be required for PINK1 cleavage in *Drosophila* [31], our results suggest that PARL, its mammalian ortholog, is not required for PINK1 cleavage in mammalian cells. This situation is similar to that of OPA1, which also requires Rhomboid-7 for cleavage in *Drosophila*, but does not require PARL for cleavage in mammalian cells [48].

In addition, determining how PINK1 cleavage is modulated by membrane potential will require further study. The protease itself may be sensitive to membrane potential and/or the PINK1 cleavage site may be available to the protease only in the presence of a membrane potential. Alternatively, the regulation of PINK1 cleavage by membrane potential may be indirect. That inhibition of PINK1 cleavage by mitochondrial depolarization up-regulates the PINK1/Parkin mitophagy pathway also raises the possibility that inhibitors of PINK1's protease might up-regulate the pathway and have some therapeutic benefit.

Our results suggest that PINK1 induces Parkin recruitment to a particular subset of mitochondria, following its accumulation, and there are several models for how PINK1 might induce Parkin recruitment. In the simplest, as PINK1 accumulates, Parkin may be recruited to mitochondria through a direct interaction with the accumulated PINK1. In support of this model, PINK1 appears to directly bind Parkin at least in some contexts [29]. Alternatively, PINK1 may need to phosphorylate Parkin, a substrate of Parkin, or an adaptor between PINK1 and Parkin, and thereby increase Parkin's affinity for a substrate or receptor on mitochondria. Consistent with a role for phosphorylation in the activation of Parkin, we found a kinase-deficient version of PINK1 fails to rescue Parkin recruitment to mitochondria in PINK1 null MEFs (even though PINK1 KD appears to be processed identically to wild-type PINK1). We were unable to replicate findings suggesting that phosphorylation of two threonines in a conserved region of Parkin is sufficient to induce Parkin recruitment to mitochondria [27], but it is possible that Parkin may be phosphorylated by PINK1 elsewhere. If direct phosphorylation is sufficient to induce Parkin recruitment to mitochondria, however, it seems difficult to explain how Parkin can be targeted to a particular subset of mitochondria, as appears to occur in cells with bioenergetically diverse populations of mitochondria [21].

Mutations in Parkin and PINK1 are inherited primarily in a recessive manner, and loss of their function is thought to cause early-onset Parkinson disease. We find that patient mutations in PINK1 and Parkin disrupt the PINK1/Parkin mitochondrial turnover pathway at distinct steps, consistent with the potential relevance of this pathway for the development of Parkinson disease.

Mutations in Parkin's UBL or its deletion caused a moderate deficit in Parkin recruitment to depolarized mitochondria and induction of mitophagy. That deletion of the UBL only partially inhibited the recruitment of Parkin to mitochondria suggests that whereas this domain promotes the recruitment of Parkin to mitochondria, it is not absolutely necessary for recruitment or subsequent mitophagy. The UBL likely promotes recruitment of Parkin through interaction with a protein containing a ubiquitin-binding domain, as mutating residue isoleucine 44, which is critical for the interaction between UBLs and UBDs [47], to alanine resulted in a recruitment deficit similar to that caused by deletion of the UBL domain. The disease-causing mutations R42P, which causes global unfolding by NMR [49], and A46P lie on either ends of the beta-pleated sheet containing I44A, suggesting that these mutations may inhibit Parkin recruitment by disrupting the interaction between Parkin and UBD-containing proteins (Figure S7A and S7B).

Mutations in key cysteine residues in the RBR domain or deletion of RING2, which is responsible for Parkin's ubiquitin ligase activity, severely disrupt both the recruitment of Parkin to mitochondria and its induction of mitophagy. Interestingly, the R275W mutation in RING1 of Parkin causes only a minor disturbance of Parkin recruitment to depolarized mitochondria but severely disrupts mitophagy, suggesting that recruitment and mitophagy can be experimentally disassociated.

The R275W polymorphism in Parkin and the G411S polymorphism in PINK1 have only been identified as heterozygous polymorphisms in cases of Parkinson disease [42],[50]. For this reason, the pathogenicity of these polymorphisms has been a matter of controversy. Our results show that the R275W Parkin mutation, which affects a highly conserved arginine residue, causes a significant loss of Parkin function in our mitophagy assay. This is consistent with in vivo data in *Drosophila melanogaster*, demonstrating that Parkin R275W, unlike wild-type Parkin, fails to compensate for loss of endogenous Parkin. By contrast, we found that PINK1 containing the G411S polymorphism, which is conserved in vertebrates, but not invertebrates, could compensate for loss of endogenous PINK1, consistent with the view that PINK1 G411S may be a natural variant and not a disease-causing mutation.

The stringent dependence of Parkin recruitment on PINK1 under depolarizing conditions is a little surprising given that, when overexpressed, Parkin can partially compensate for PINK1 loss in *Drosophila* and in mammalian cells [11]–[13],[20]. How Parkin overexpression compensates for PINK1 loss is not known, but there are several possible explanations. First, there may be mechanisms independent of PINK1 and depolarization that can recruit Parkin to dysfunctional mitochondria. Alternatively, Parkin may serve other functions in the cell that are independent of PINK1 and protect against mitochondrial dysfunction indirectly; or Parkin may function to some degree upon overexpression independently of mitochondrial docking, perhaps effecting mitophagy or other mitochondrial changes from the cytosolic compartment.

Stable loss or knockdown of PINK1 in mammalian cellular models and mice leads to a number of mitochondria-related abnormalities. Mitochondria in these cells or tissues exhibit electron transport chain (ETC) dysfunction, diminished membrane potential, increased reactive oxygen species production, mitochondrial fragmentation, and calcium dysregulation, among other abnormalities [20],[32],[34],[51]. Although some of these abnormalities may be a reversible consequence of others—for instance, mitochondrial fragmentation may be due to low membrane potential [34], and ETC dysfunction and decreased membrane potential may be, in part, a functional consequence of calcium dysregulation [51]—other abnormalities may be due to irreversible dysfunction of specific mitochondrial proteins or protein complexes. For instance, Complex I and the putative Na^+^/Ca2^+^ transporter seem to be dysfunctional in cultured cells following PINK1 knockdown [51], whereas Complex I and II appear to be dysfunctional in the striatum of mice lacking PINK1 [16].

Although the proximate cause of these abnormalities in PINK1 null cells remains obscure, one explanation may be the failure of PINK1/Parkin pathway to eliminate oxidatively damaged mitochondria, which accumulate over time as a natural consequence of metabolism and other cellular stresses. That Parkin null cells and tissues appear to share some of the same mitochondrial defects as PINK1 null cells and tissues supports the view that these abnormalities may be due to loss of a common PINK1/Parkin pathway [17],[18]. We cannot rule out that PINK1 may actively prevent mitochondrial damage and dysfunction, in addition to its signaling role in the PINK1/Parkin pathway. PINK1's interaction with HtrA2/OMI, for instance, appears to be independent of Parkin function in *Drosophila* [31],[52],[53].

Loss of PINK1 and Parkin affects some cell populations, like substantia nigral neurons, more than others, even though PINK1 and Parkin appear to be more widely expressed. Why some tissues are more vulnerable to loss of PINK1/Parkin than others is unclear, but it may relate to the degree of damage mitochondria sustain within that tissue (e.g., mitochondria in the SN are subject to greater oxidative stress than those in other neural tissues [2]); the existence of redundant mitophagy pathways (e.g., mammalian tissues may contain pathways orthologous to those recently identified in yeast [54],[55]); the ability of the tissue to mitigate the damage by other means (a tissue composed of mitotic cells may be able to manage mitochondrial damage through cellular turnover rather than mitochondrial turnover); and mitochondrial demand within a particular tissue (neurons have high, local metabolic demands, and dopaminergic neurons are subject to especially high calcium fluxes that need to be buffered by mitochondria [56]). Some or all of these factors may contribute to the special reliance of SN neurons on PINK1 and Parkin.

PINK1 and Parkin are a significant cause of autosomal recessive parkinsonism and have been genetically linked to a pathway that protects against progressive mitochondrial damage and dysfunction. We have found that PINK1 levels and subsequently Parkin recruitment to mitochondria are dramatically regulated by the bioenergetic state of individual mitochondria, and that this unique regulation may allow PINK1 and Parkin to promote the selective and efficient turnover of mitochondria that have become damaged. Loss of PINK1 or Parkin function due to pathogenic mutations can disrupt this mitochondrial turnover pathway which may lead to the accumulation of dysfunctional mitochondria in vulnerable tissues—with a resultant increase in oxidative stress, depression of metabolism, and, eventually, accelerated cell death, which has been observed in *Drosophila* and, to a lesser extent, in mouse models of the disease [11]–[17]. Together, these findings provide a biochemical explanation for the genetic epistasis between PINK1 and Parkin observed in *Drosophila*, and support a novel, testable model of how loss of PINK1 and Parkin function may lead to autosomal recessive parkinsonism.

## Materials and Methods

### Cell Culture

HeLa YFP-Parkin, E18 Rat cortical neurons, PINK1^+/+^ SV40-transformed MEF cells, PINK1^−/−^ SV40-transformed MEF, M17 neuroblastoma control shRNA, M17 neuroblastoma PINK1, Mfn1/2^−/−^ MEF, and Parl^−/−^ MEF cell lines have been described previously [21],[25],[29],[33],[57],[58]. PINK1^+/+^ and PINK1^−/−^ primary MEFs were isolated from embryos using a standard protocol [16]. Parkin^+/+^- and Parkin^−/−^-transformed MEFs were created by isolation of primary cells from embryos of B6.129S4-*Park2^tm1Shn^*/J mice (Jackson Labs), using a standard protocol [16], followed by retroviral transduction of SV40 (Applied Biological Materials). YFP-Parkin, YFP-Parkin mutants, mCherry-Parkin, PINK1-YFP, PINK1KD-YFP, PINK1 Δ1-110-YFP, and Opa3-PINK1 Δ1-110-YFP are in C1 or N1 Clontech vectors. PINK1WT-V5, PINK1KD-V5, and PINK1 Δ1-156-V5 are in pDest40 vector (Invitrogen). PINK1 patient mutations are in the pLenti-V5 vector (Invitrogen). PINK1-myc is in a pCMBTNT vector (Promega). The PARL shRNA construct targeting (5′-CCAACTTGGAGCTTCTAGTAAGTTCTCTACTAGAAGCTCCAAGTTGG-3′) is in the pSuper-GFP vector (OligoEngine). To make FRB-PINK1 (111–581)-YFP and Tom20 (1–33)-FKBP, PCR fragments containing PINK1 (111–581)-YFP and Tom20 (1–33) were cloned into the BamHI site of the pC_4_-R_H_E vector and the EcoRI and XbaI sites of pC_4_M-F2E vectors, respectively (ARIAD Pharmaceuticals). The rapamycin analog AP21967 was obtained from ARIAD Pharmaceuticals.

### Confocal Microscopy

Confocal microscopy of fixed samples, scoring of Parkin recruitment and Parkin-induced mitophagy, and live-cell imaging were performed as described previously [21]. Experiments in Mfn1/2 null cells were performed as described previously, with minor modifications, as described in the supplemental materials and methods (Text S1) [21].

### Immunoblotting and Immunocytochemistry

For PINK1 experiments, cells were fractionated using the Mitochondria Isolation Kit (Pierce), according to manufacturer's specifications, with slight modifications, as described in the supplemental methods (Text S1). To isolate integral membrane proteins, membrane fractions obtained as above were carbonate extracted with 0.1 M Na_2_CO_3_ fresh cold buffer, and membranes were pelleted, as described in the supplemental materials and methods (Text S1). For Parkin experiments, cells were fractionated as described previously, with minor modifications, as described in the supplemental materials and methods (Text S1) [21]. The protease protection assay was performed as described previously [22]. Cells were fixed and immunostained as described previously [21]. The following primary antibodies were used: anti-Parkin (PRK8) monoclonal (Santa Cruz Biotechnology), anti-Tom20 polyclonal (Santa Cruz Biotechnology.), anti-cytochrome c monoclonal (BD Biosciences), anti-PINK1 polyclonal (Novus Biologicals), anti-VDAC monoclonal (Calbiochem), anti-GAPDH polyclonal (Sigma-Aldrich), anti-Tubulin monoclonal (Sigma-Aldrich), anti-V5 monoclonal (Invitrogen), anti-GFP polyclonal (Invitrogen), anti-TIM23 monoclonal (BD Biosciences), and anti-Hsp60 monoclonal (Stressgen).

### Quantitative RT-PCR

qRT-PCR of PINK1 mRNA levels was performed as described in detail previously [34].

## Supporting Information

Figure S1 **Mitochondrial PINK1 accumulates on the outer mitochondrial membrane following mitochondrial depolarization.** (A) HeLa cells treated with 1 µM of valinomycin without serum at time point 0 were fractionated, and carbonate extracted. The carbonate extracted pellet, which is enriched for proteins integral to mitochondria, was run on SDS gels and immunoblotted for endogenous PINK1 and mitochondrial protein VDAC. (B) HeLa cells stably expressing YFP-Parkin were treated with 2 µM of CCCP without serum at time point 0 and fractionated. The mitochondria-rich membrane fraction (lanes 1 and 2) and the cytosolic enriched postmembrane fraction (lanes 4–9) were run on SDS gels and immunoblotted for PINK1, tubulin, and VDAC. (C) PINK1^−/−^ MEFs transfected with PINK1-myc or left untransfected were treated with 2 µM CCCP without serum for 3 h and fractionated. Mitochondrial-rich membrane fraction was run on SDS gels and immunoblotted for PINK1 and VDAC. (D) HeLa cells transfected with PINK1-YFP or a kinase-deficient version of PINK1 (PINK1KD-YFP) were treated as in (B). Whole-cell lysates were run on SDS gels and immunoblotted for PINK1 and tubulin. Arrow indicates the predicted molecular weight (MW) of full-length PINK1-YFP. (E) HeLa cells stably expressing YFP-Parkin were treated with 10 µM CCCP for 3 h and fractionated. The mitochondria-enriched membrane fraction was aliquoted. Each aliquot was treated with 0 to 100 µg/ml protease K and immunoblotted for endogenous PINK1, the outer membrane protein TOM20, the inner membrane protein Tim23, and matrix protein Hsp60.
(2.72 MB TIF)

Figure S2 **PINK1 accumulates independently of PARL and Parkin expression.** (A) HeLa cells cotransfected with PARL-Flag and either PARL shRNA or control shRNA were depolarized with 10 µM CCCP for 3 h. Whole-cell lysates were run on SDS gels and immunoblotted with antibodies against the N-terminus of PARL, the C-terminus of PARL, and tubulin. (B) HeLa cells mock transfected or transfected with shRNA PARL were treated with DMSO or CCCP for 3 h and fractionated. The mitochondria-enriched membrane fraction (left) and whole-cell lysates (right) were run on SDS gels and immunoblotted for PINK1, the C-terminus of PARL, VDAC, and/or tubulin. (C) Wild-type or PARL-null MEFs were transfected with PINK1-V5, treated as in (B), and fractionated. The mitochondria-enriched heavy membrane fraction was run on SDS gels and immunoblotted for PINK1, the N-terminus of PARL, the C-terminus of PARL, and Hsp60. (D) Untransfected HeLa cells or HeLa cells stably expressing YFP-Parkin (HeLa/Parkin) were treated with DMSO or 2 µM CCCP without serum for 1 h. Whole-cell lysates (WCL) were run on SDS gels and immunoblotted for endogenous PINK1 and tubulin. (E) Parkin^+/+^ or Parkin^−/−^ MEFs transfected with PINK1-myc were treated with 2 µM CCCP in the absence of serum and fractionated. The mitochondria-rich membrane fraction was immunoblotted for PINK1 and VDAC. Scale bars in all images represent 10 µm.
(2.32 MB TIF)

Figure S3 **PINK1 is required for Parkin recruitment to mitochondria.** (A) SV40-transformed PINK1^−/−^ MEFs cotransfected with YFP-Parkin (green) and vector, PINK1, or PINK1 KD were treated with 20 µM CCCP for 3 h. Cells were immunostained for Tom20 (red). (B) M17 neuroblastoma cells stably transduced with control shRNA or PINK1 shRNA and transfected with YFP-Parkin were treated with DMSO or 10 µM CCCP for 3 h and fractionated into postnuclear supernatant (PNS), mitochondria-rich heavy membrane fraction (HMF), and supernatant (Sup). Fractions were run on SDS gels and immunoblotted for Parkin, PINK1, and VDAC. Scale bars in images represent 10 µm.
(1.39 MB TIF)

Figure S4 **Increased expression of PINK1 on the outer mitochondrial membrane is sufficient for Parkin recruitment.** (A) A schematic demonstrating the construction of PINK1-YFP, FRB-PINK1 (111–581)-YFP, and Tom20(1–33)-FKBP. The FRB and FKBP domains heterodimerize in the presence of the rapamycin analog AP21967 if they are in the same compartment. (B) HeLa cell were transfected with PINK1 (111–581)-YFP and Tom20 (1–33)-FKBP, and imaged live. The rapamycin analog, AP21967 (250 nM), was added at time point 0. (C) Confocal image depicting the localization of FRB-PINK1 (111–581)-YFP (green) cotransfected with Tom20 (1–33)-FKBP following treatment with vehicle or 250 nM of AP21967 for 30 min. Mitochondria are labeled with the potentiometric dye TMRE (red). (D) Confocal image depicting the localization of FRB-PINK1 (111–581)-YFP (green) and mCherry-Parkin (red) following treatment with vehicle or 250 nM of AP21967 for 8 h. Scale bars in all images represent 10 µm.
(2.16 MB TIF)

Figure S5 **Putative PINK1 phosphorylation sites on Parkin, T175, and T217 are not sufficient for Parkin recruitment.** (A) Alignment of highly conserved Parkin unique region/domain containing T175 and T217. Arrows on top show positions of threonines 175 and 217 and the disease-causing mutation C212. Brackets on the bottom of the alignment point to the conserved cysteine and histidine residues forming putative zinc-binding sites I and II of the RING0 domain. (B) HeLa cells were transfected with YFP-Parkin (white) containing the indicated point mutations and treated with DMSO or CCCP for 1 h. Mitochondria were labeled with anti-Tom20 antibody. (C) Colocalization between YFP-Parkin and mitochondria in (A) was scored for >150 cells/condition in three or more independent experiments. Scale bars in all images represent 10 µm.
(2.17 MB TIF)

Figure S6 **Disease-causing mutations in PINK1 do not affect PINK1-induced accumulation.** (A) HeLa cells stably expressing Parkin transfected with the indicated V5-tagged PINK1 constructs were treated with DMSO or 2 µM CCCP in serum-free medium. Whole-cell lysates were run on SDS gels and immunoblotted for PINK1, the V5 tag, and tubulin.
(0.67 MB TIF)

Figure S7 **Mutations in Parkin's ubiquitin-like domain (UBL) partially disrupt Parkin recruitment to mitochondria.** (A) Alignment of part of the UBL amino acid sequences from orthologous Parkin proteins. An asterisk (*) indicates position of patient mutations (R42P and R46) and engineered mutation (I44A) examined below. Red box indicates position of beta-pleated sheet containing I44, a key residue for interactions between UBL domains and ubiquitin-binding domains. (B) Structure of UBL (PDB 1IYF) with position of patient mutations (R42P and A46P) highlighted in blue and position of engineered mutation I44A highlighted in red. (C and D) HeLa cells transfected with YFP-Parkin containing indicated mutations (green) and treated with CCCP for 1 h (C) or 24 h (D). Mitochondria were labeled with Tom20 antibody (red). (E) Colocalization between YFP-Parkin and mitochondria in (C) scored for ≥150 cells/condition in three or more independent experiments. (F) Percentage of cells with no mitochondria transfected and treated as described in (D) scored for ≥150 cells/condition in three or more independent experiments. Scale bars in all images represent 10 µm.
(3.70 MB TIF)

Figure S8 **Mutations in Parkin disrupt Parkin recruitment and/or Parkin-mediated mitophagy.** (A) HeLa cells transfected with YFP-Parkin (green) containing the indicated missense mutations were treated with 10 µM CCCP in serum for 24 h were scored for Parkin colocalizing with mitochondria in ≥150 cells/condition in three or more independent experiments. Mitochondria were immunostained for Tom20 (red). (B) Confocal images representing (A). (C) HeLa cells transfected with YFP-Parkin R275W (green) were imaged live. CCCP (10 µM) was added at time point 0. (D) HeLa cells transfected with the indicated constructs were treated with DMSO or 10 µM CCCP for 24 h and scored for the percentage of cells with visible aggregates in ≥150 cells/condition in three or more independent experiments. (E) HeLa cells transfected with YFP-Parkin WT (green) or YFP-Parkin R275W (green) were treated with DMSO or CCCP for 1 h and imaged live. Mitochondria were stained with the potentiometric dye TMRE (red). Scale bars in all images represent 10 µm.
(2.40 MB TIF)

Figure S9 **Model depicting regulation of PINK1 stability on healthy and dysfunctional mitochondria by membrane potential.** On healthy mitochondria PINK1 is constitutively imported, proteolytically cleaved into a cytosolic form, and degraded by the proteasome, resulting in low levels of mitochondrial PINK1. On damaged mitochondria with low membrane potentials (ΔΨ), however, PINK1 cleavage is blocked, leading to accumulation of mitochondrial PINK1 on the dysfunctional mitochondria. Accumulated PINK1 recruits Parkin to damaged mitochondria, which Parkin marks, likely by ubiquitination, for autophagic degradation.
(0.39 MB TIF)

Text S1 **Supplemental experimental methods.**
(0.03 MB DOC)

Video S1 **PINK1-YFP accumulates on mitochondria.** HeLa cells transiently transfected with PINK1-YFP (green) were incubated in CO_2_-independent medium (Gibco) for 1 h and then treated with 10 µM CCCP at time point 0. Images of cells were taken every 1 min for 40 min, using a PerkinElmer UltraView LCI (Live Cell Imaging) System at 35°C.
(2.67 MB MOV)

Video S2 **mCherry-Parkin is recruited to mitochondria.** HeLa cells transiently transfected with mCherry-Parkin (red) + empty vector in a 1∶1 ratio were incubated in CO_2_-independent medium (Gibco) for 1 h and then treated with 10 µM CCCP at time point 0. Images of cells were taken every 1 min for 68 min, using a PerkinElmer UltraView LCI (Live Cell Imaging) System at 35°C.
(2.00 MB MOV)

Video S3 **Kinetics of mCherry-Parkin recruitment to mitochondria are enhanced by exogenous PINK1 expression.** HeLa cells transiently transfected with mCherry-Parkin (red) + PINK1-YFP in a 1∶1 ratio were incubated in CO_2_-independent medium (Gibco) for 1 h and then treated with 10 µM CCCP at time point 0. Images of cells were taken every 1 min for 51 min, using a PerkinElmer UltraView LCI (Live Cell Imaging) System at 35°C.
(4.11 MB MOV)

## Abbreviations

mtDNA: mitochondrial DNA
MTG: Mitotracker Green
shRNA: short hairpin RNA
SD: standard deviation
SN: substantia nigra
