# Supplementary figures and images for: PINK1 Is Selectively Stabilized on Impaired Mitochondria to Activate Parkin
Source: PLoS Biol. 2010 Jan 26;8(1):e1000298. doi: 10.1371/journal.pbio.1000298 (PMC2811155; doi:10.1371/journal.pbio.1000298)

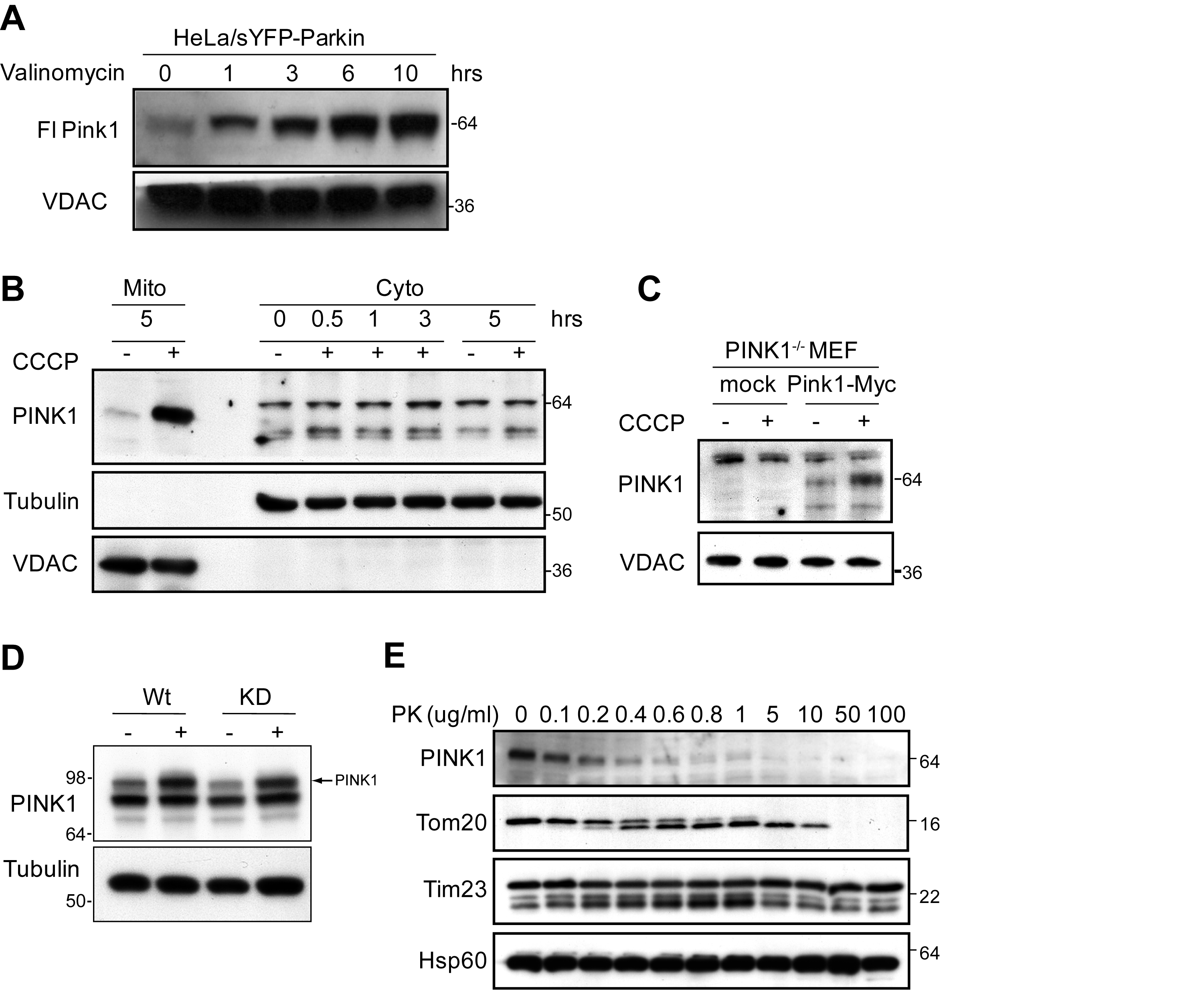

Supplement: Figure S1 — Mitochondrial PINK1 accumulates on the outer mitochondrial membrane following mitochondrial depolarization. (A) HeLa cells treated with 1 µM of valinomycin without serum at time point 0 were fractionated, and carbonate extracted. The carbonate extracted pellet, which is enriched for proteins integral to mitochondria, was run on SDS gels and immunoblotted for endogenous PINK1 and mitochondrial protein VDAC. (B) HeLa cells stably expressing YFP-Parkin were treated with 2 µM of CCCP without serum at time point 0 and fractionated. The mitochondria-rich membrane fraction (lanes 1 and 2) and the cytosolic enriched postmembrane fraction (lanes 4–9) were run on SDS gels and immunoblotted for PINK1, tubulin, and VDAC. (C) PINK1−/− MEFs transfected with PINK1-myc or left untransfected were treated with 2 µM CCCP without serum for 3 h and fractionated. Mitochondrial-rich membrane fraction was run on SDS gels and immunoblotted for PINK1 and VDAC. (D) HeLa cells transfected with PINK1-YFP or a kinase-deficient version of PINK1 (PINK1KD-YFP) were treated as in (B). Whole-cell lysates were run on SDS gels and immunoblotted for PINK1 and tubulin. Arrow indicates the predicted molecular weight (MW) of full-length PINK1-YFP. (E) HeLa cells stably expressing YFP-Parkin were treated with 10 µM CCCP for 3 h and fractionated. The mitochondria-enriched membrane fraction was aliquoted. Each aliquot was treated with 0 to 100 µg/ml protease K and immunoblotted for endogenous PINK1, the outer membrane protein TOM20, the inner membrane protein Tim23, and matrix protein Hsp60. (2.72 MB TIF) [file pbio.1000298.s001.tif]

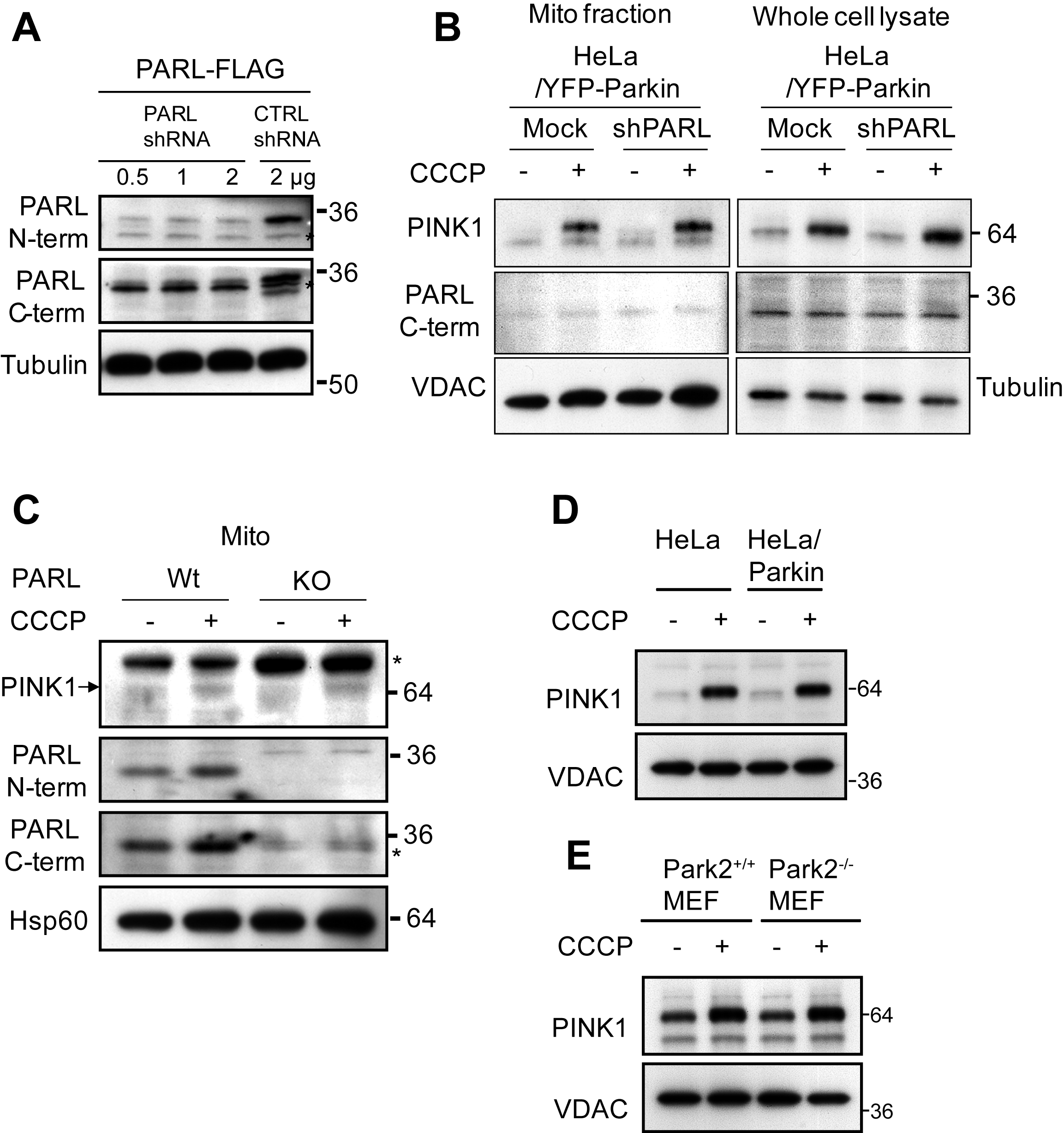

Supplement: Figure S2 — PINK1 accumulates independently of PARL and Parkin expression. (A) HeLa cells cotransfected with PARL-Flag and either PARL shRNA or control shRNA were depolarized with 10 µM CCCP for 3 h. Whole-cell lysates were run on SDS gels and immunoblotted with antibodies against the N-terminus of PARL, the C-terminus of PARL, and tubulin. (B) HeLa cells mock transfected or transfected with shRNA PARL were treated with DMSO or CCCP for 3 h and fractionated. The mitochondria-enriched membrane fraction (left) and whole-cell lysates (right) were run on SDS gels and immunoblotted for PINK1, the C-terminus of PARL, VDAC, and/or tubulin. (C) Wild-type or PARL-null MEFs were transfected with PINK1-V5, treated as in (B), and fractionated. The mitochondria-enriched heavy membrane fraction was run on SDS gels and immunoblotted for PINK1, the N-terminus of PARL, the C-terminus of PARL, and Hsp60. (D) Untransfected HeLa cells or HeLa cells stably expressing YFP-Parkin (HeLa/Parkin) were treated with DMSO or 2 µM CCCP without serum for 1 h. Whole-cell lysates (WCL) were run on SDS gels and immunoblotted for endogenous PINK1 and tubulin. (E) Parkin+/+ or Parkin−/− MEFs transfected with PINK1-myc were treated with 2 µM CCCP in the absence of serum and fractionated. The mitochondria-rich membrane fraction was immunoblotted for PINK1 and VDAC. Scale bars in all images represent 10 µm. (2.32 MB TIF) [file pbio.1000298.s002.tif]

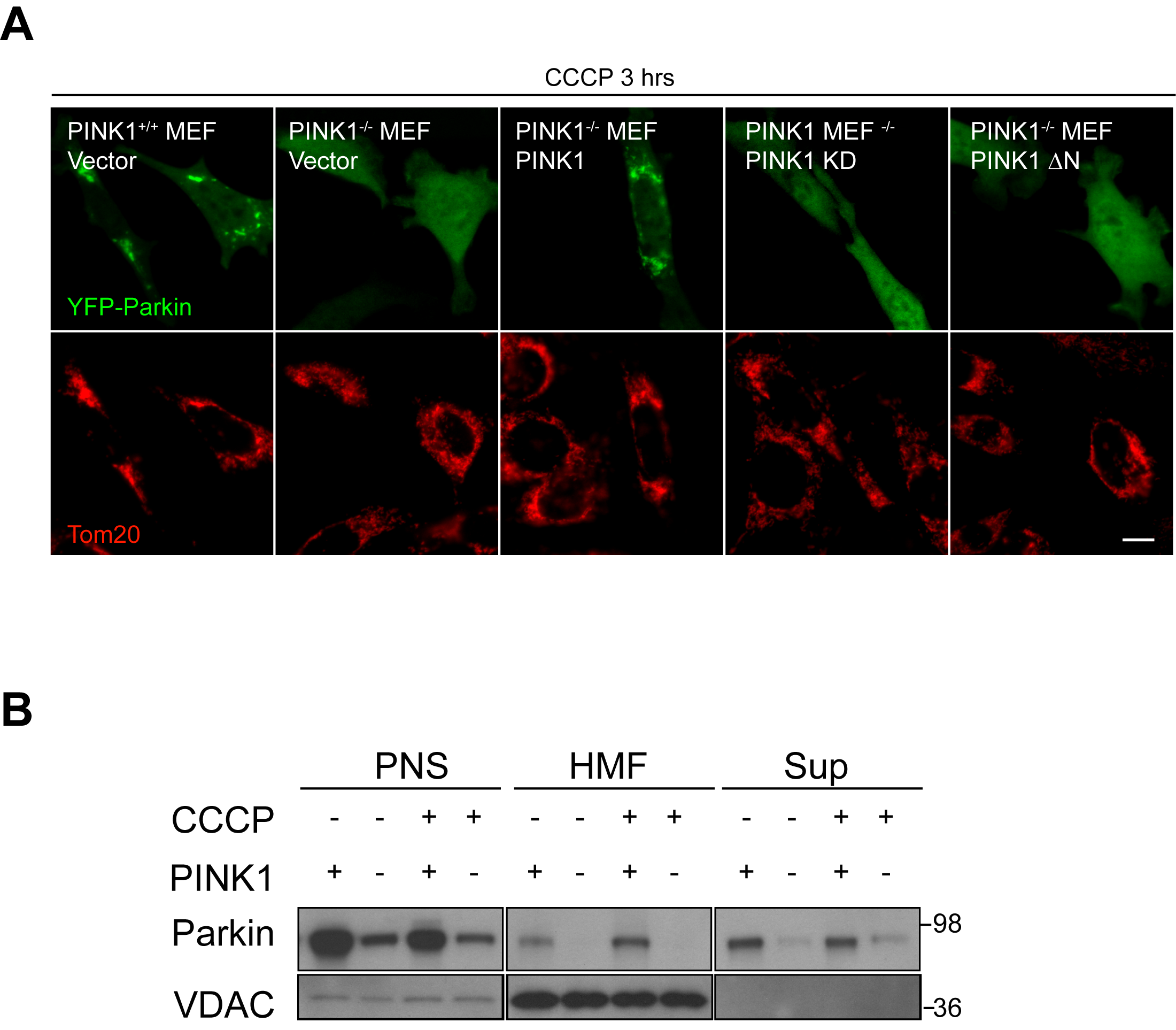

Supplement: Figure S3 — PINK1 is required for Parkin recruitment to mitochondria. (A) SV40-transformed PINK1−/− MEFs cotransfected with YFP-Parkin (green) and vector, PINK1, or PINK1 KD were treated with 20 µM CCCP for 3 h. Cells were immunostained for Tom20 (red). (B) M17 neuroblastoma cells stably transduced with control shRNA or PINK1 shRNA and transfected with YFP-Parkin were treated with DMSO or 10 µM CCCP for 3 h and fractionated into postnuclear supernatant (PNS), mitochondria-rich heavy membrane fraction (HMF), and supernatant (Sup). Fractions were run on SDS gels and immunoblotted for Parkin, PINK1, and VDAC. Scale bars in images represent 10 µm. (1.39 MB TIF) [file pbio.1000298.s003.tif]

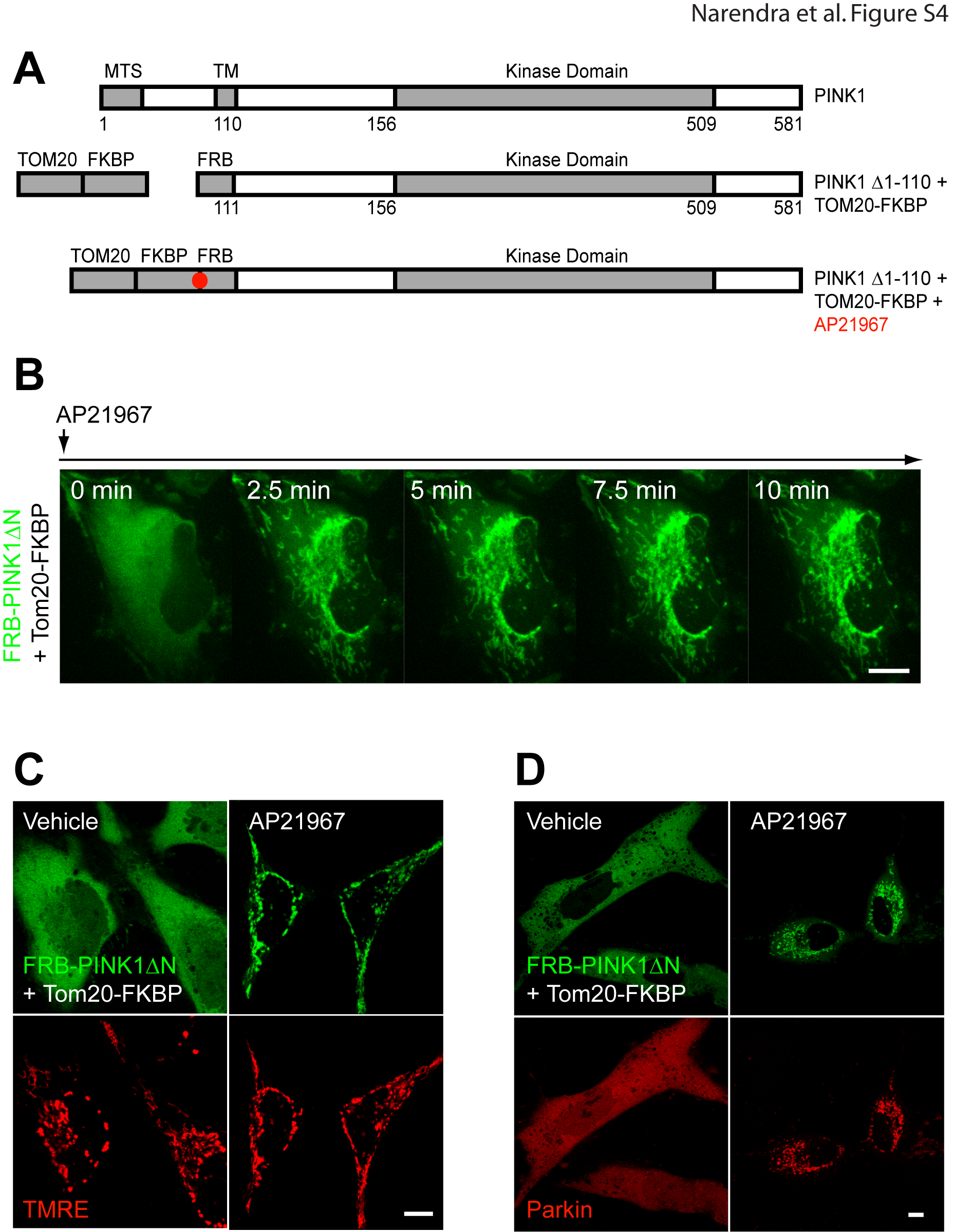

Supplement: Figure S4 — Increased expression of PINK1 on the outer mitochondrial membrane is sufficient for Parkin recruitment. (A) A schematic demonstrating the construction of PINK1-YFP, FRB-PINK1 (111–581)-YFP, and Tom20(1–33)-FKBP. The FRB and FKBP domains heterodimerize in the presence of the rapamycin analog AP21967 if they are in the same compartment. (B) HeLa cell were transfected with PINK1 (111–581)-YFP and Tom20 (1–33)-FKBP, and imaged live. The rapamycin analog, AP21967 (250 nM), was added at time point 0. (C) Confocal image depicting the localization of FRB-PINK1 (111–581)-YFP (green) cotransfected with Tom20 (1–33)-FKBP following treatment with vehicle or 250 nM of AP21967 for 30 min. Mitochondria are labeled with the potentiometric dye TMRE (red). (D) Confocal image depicting the localization of FRB-PINK1 (111–581)-YFP (green) and mCherry-Parkin (red) following treatment with vehicle or 250 nM of AP21967 for 8 h. Scale bars in all images represent 10 µm. (2.16 MB TIF) [file pbio.1000298.s004.tif]

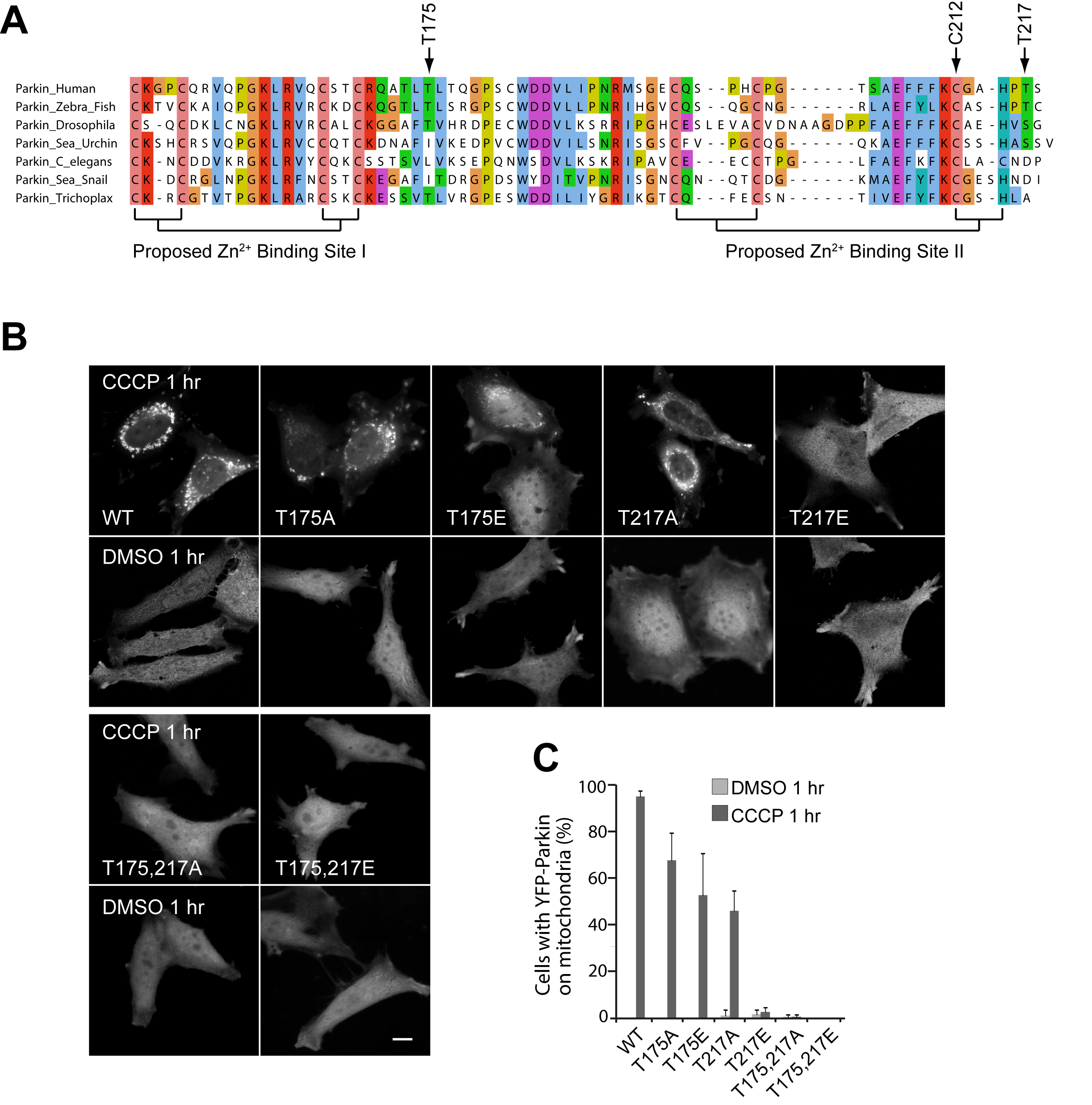

Supplement: Figure S5 — Putative PINK1 phosphorylation sites on Parkin, T175, and T217 are not sufficient for Parkin recruitment. (A) Alignment of highly conserved Parkin unique region/domain containing T175 and T217. Arrows on top show positions of threonines 175 and 217 and the disease-causing mutation C212. Brackets on the bottom of the alignment point to the conserved cysteine and histidine residues forming putative zinc-binding sites I and II of the RING0 domain. (B) HeLa cells were transfected with YFP-Parkin (white) containing the indicated point mutations and treated with DMSO or CCCP for 1 h. Mitochondria were labeled with anti-Tom20 antibody. (C) Colocalization between YFP-Parkin and mitochondria in (A) was scored for >150 cells/condition in three or more independent experiments. Scale bars in all images represent 10 µm. (2.17 MB TIF) [file pbio.1000298.s005.tif]

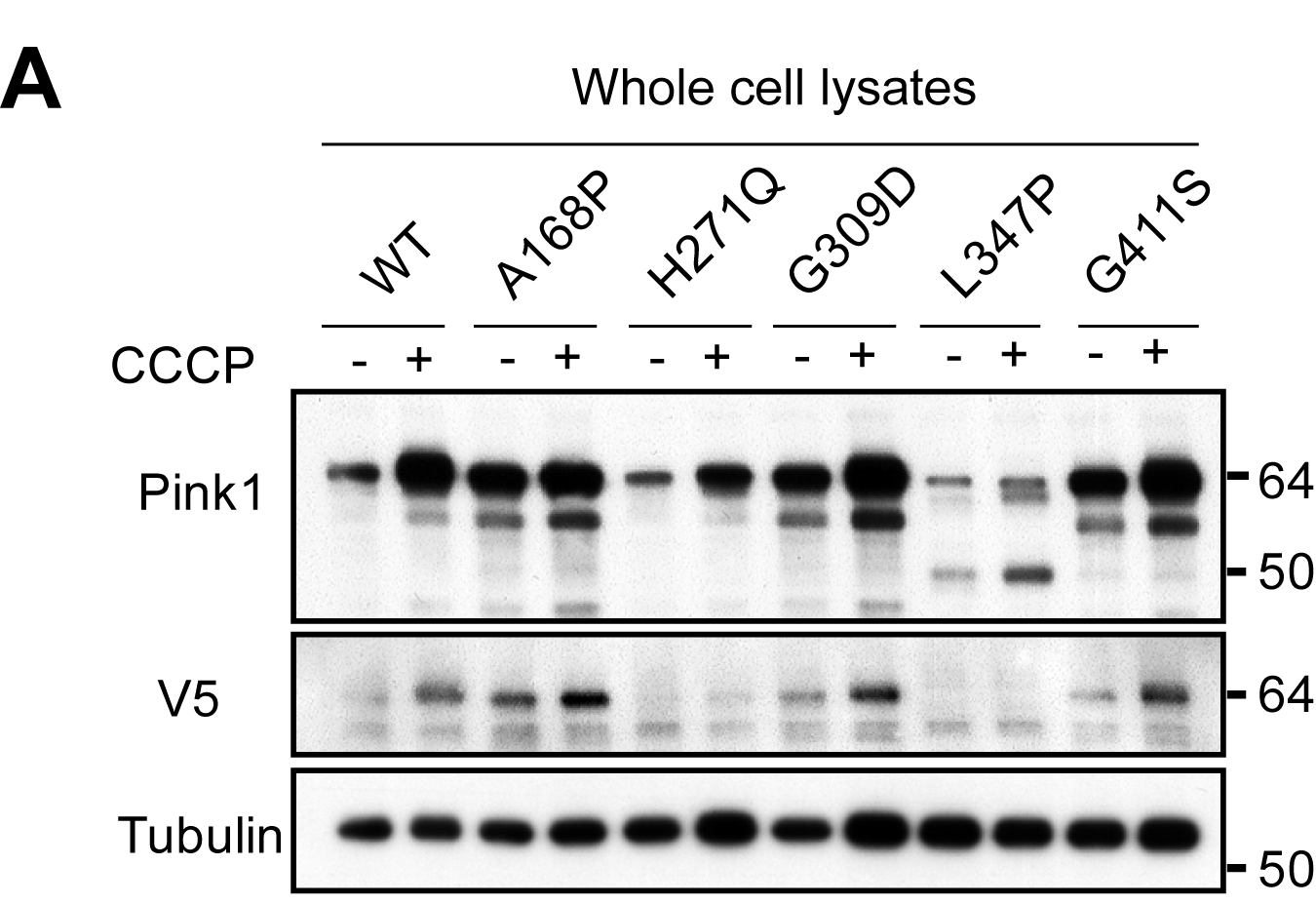

Supplement: Figure S6 — Disease-causing mutations in PINK1 do not affect PINK1-induced accumulation. (A) HeLa cells stably expressing Parkin transfected with the indicated V5-tagged PINK1 constructs were treated with DMSO or 2 µM CCCP in serum-free medium. Whole-cell lysates were run on SDS gels and immunoblotted for PINK1, the V5 tag, and tubulin. (0.67 MB TIF) [file pbio.1000298.s006.tif]

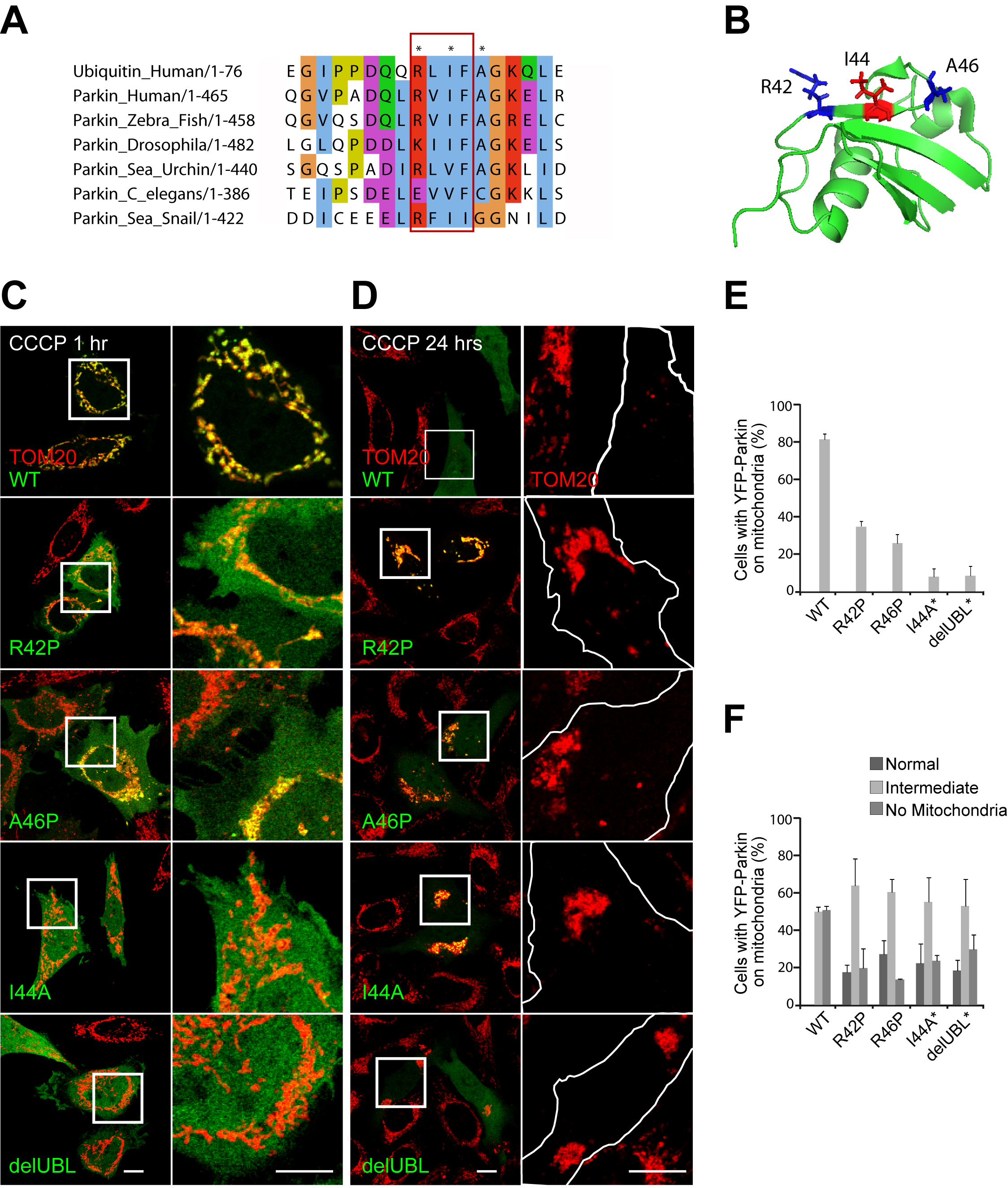

Supplement: Figure S7 — Mutations in Parkin's ubiquitin-like domain (UBL) partially disrupt Parkin recruitment to mitochondria. (A) Alignment of part of the UBL amino acid sequences from orthologous Parkin proteins. An asterisk (*) indicates position of patient mutations (R42P and R46) and engineered mutation (I44A) examined below. Red box indicates position of beta-pleated sheet containing I44, a key residue for interactions between UBL domains and ubiquitin-binding domains. (B) Structure of UBL (PDB 1IYF) with position of patient mutations (R42P and A46P) highlighted in blue and position of engineered mutation I44A highlighted in red. (C and D) HeLa cells transfected with YFP-Parkin containing indicated mutations (green) and treated with CCCP for 1 h (C) or 24 h (D). Mitochondria were labeled with Tom20 antibody (red). (E) Colocalization between YFP-Parkin and mitochondria in (C) scored for ≥150 cells/condition in three or more independent experiments. (F) Percentage of cells with no mitochondria transfected and treated as described in (D) scored for ≥150 cells/condition in three or more independent experiments. Scale bars in all images represent 10 µm. (3.70 MB TIF) [file pbio.1000298.s007.tif]

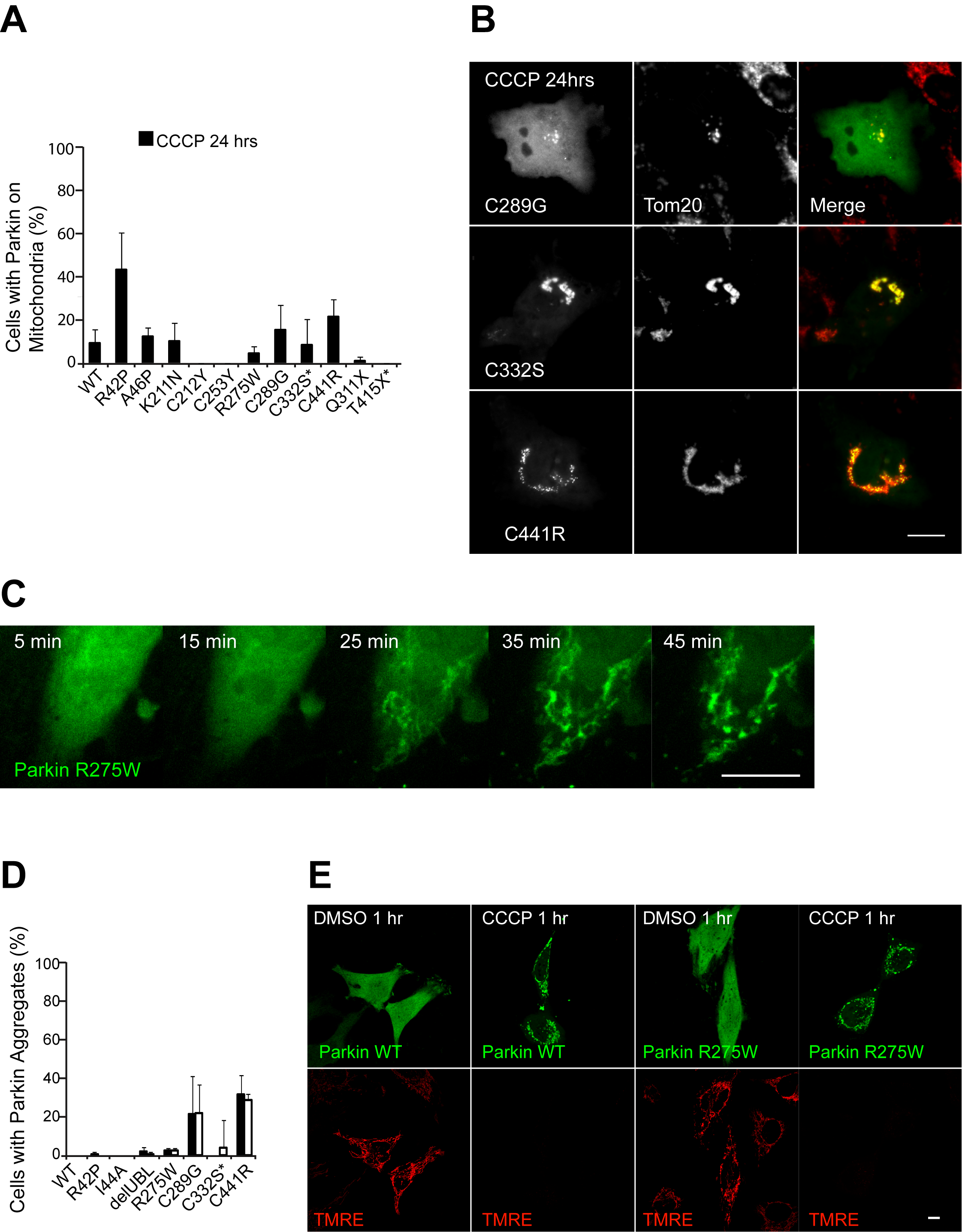

Supplement: Figure S8 — Mutations in Parkin disrupt Parkin recruitment and/or Parkin-mediated mitophagy. (A) HeLa cells transfected with YFP-Parkin (green) containing the indicated missense mutations were treated with 10 µM CCCP in serum for 24 h were scored for Parkin colocalizing with mitochondria in ≥150 cells/condition in three or more independent experiments. Mitochondria were immunostained for Tom20 (red). (B) Confocal images representing (A). (C) HeLa cells transfected with YFP-Parkin R275W (green) were imaged live. CCCP (10 µM) was added at time point 0. (D) HeLa cells transfected with the indicated constructs were treated with DMSO or 10 µM CCCP for 24 h and scored for the percentage of cells with visible aggregates in ≥150 cells/condition in three or more independent experiments. (E) HeLa cells transfected with YFP-Parkin WT (green) or YFP-Parkin R275W (green) were treated with DMSO or CCCP for 1 h and imaged live. Mitochondria were stained with the potentiometric dye TMRE (red). Scale bars in all images represent 10 µm. (2.40 MB TIF) [file pbio.1000298.s008.tif]

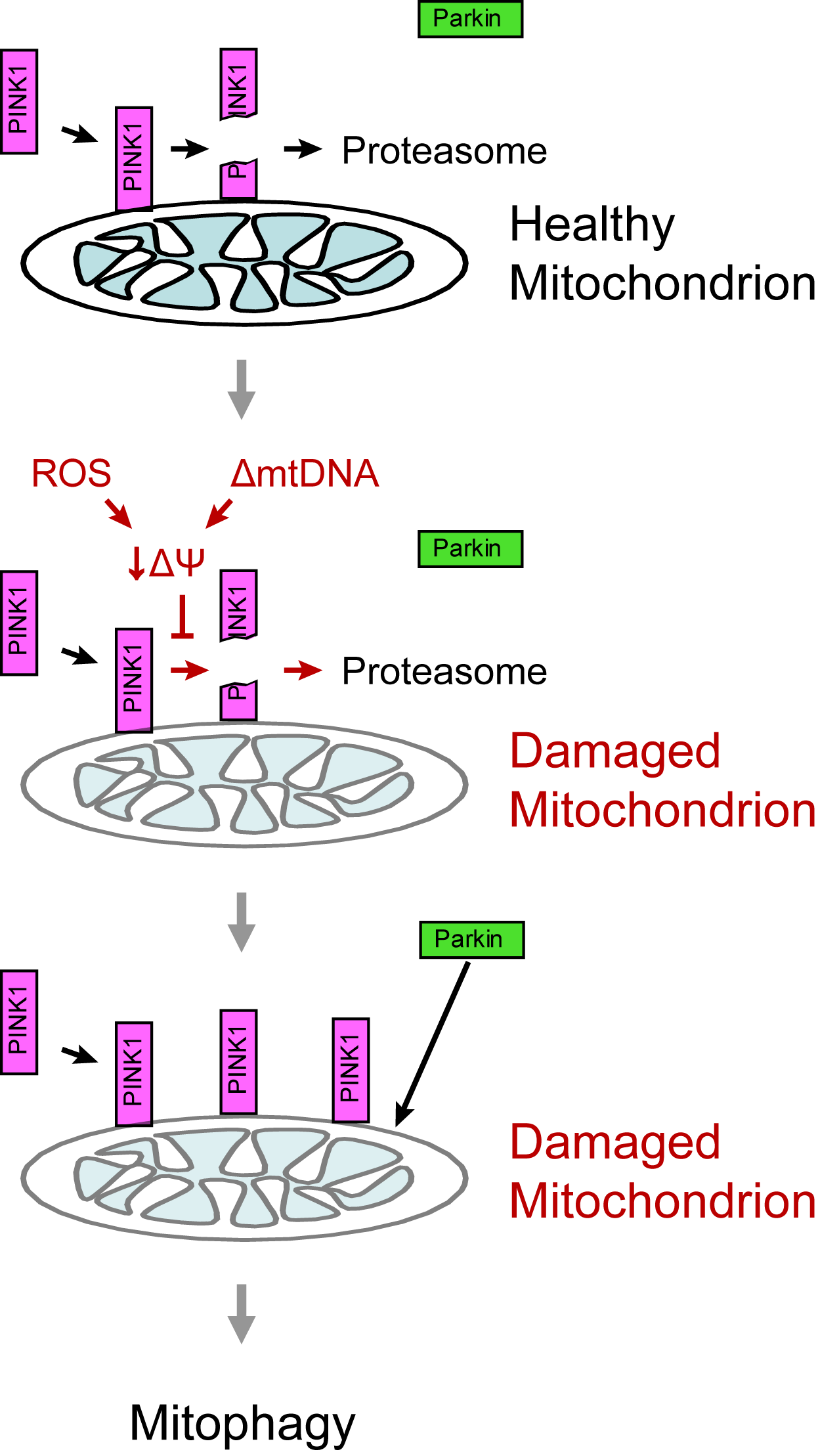

Supplement: Figure S9 — Model depicting regulation of PINK1 stability on healthy and dysfunctional mitochondria by membrane potential. On healthy mitochondria PINK1 is constitutively imported, proteolytically cleaved into a cytosolic form, and degraded by the proteasome, resulting in low levels of mitochondrial PINK1. On damaged mitochondria with low membrane potentials (ΔΨ), however, PINK1 cleavage is blocked, leading to accumulation of mitochondrial PINK1 on the dysfunctional mitochondria. Accumulated PINK1 recruits Parkin to damaged mitochondria, which Parkin marks, likely by ubiquitination, for autophagic degradation. (0.39 MB TIF) [file pbio.1000298.s009.tif]
