# Supplementary material for: PINK1 Is Selectively Stabilized on Impaired Mitochondria to Activate Parkin
Source: PLoS Biol. 2010 Jan 26;8(1):e1000298. doi: 10.1371/journal.pbio.1000298 (PMC2811155; doi:10.1371/journal.pbio.1000298)
Supplement: Text S1 — Supplemental experimental methods. (0.03 MB DOC) [file pbio.1000298.s010.doc]

Supplemental Materials and Methods

**Western Blotting.** For PINK1 experiments, we used Mitochondria Isolation Kits (Pierce) for fractionation according to the manufacturer’s instructions with minor modifications, detailed below. Briefly, cells were cultured in 100mm plates, treated as indicated in the text, washed twice with ice-cold PBS and scraped in 1 ml of ice-cold PBS. Small aliquots are preserved to prepare whole cell lysates. After removing supernatants following centrifugation, pellets are resuspended in ice-cold Mitochondria Isolation Reagent A containing protease inhibitor cocktail (GE Healthcare) and homogenized by passing 25G needle for 20 times. Mitochondria Isolation Reagent C was added and nuclear fractions were removed by centrifugation at 1,000 x G. The resulting supernatants were centrifuged again at 10,000 x G to remove cytosolic fractions. The pellets were washed once with Buffer C and used as mitochondria enriched fractions. To obtain integral membrane protein, mitochondria enriched fractions were subjected to the carbonate extraction. Shortly, mitochondrial pellets were resuspended in 50 ul of icd-cold PBS and then mixed with 500 ul of carbonate buffer (fresh cold 0.1 M Na2CO3). After 15 mins on ice, membranes were pelleted by centrifugation at 52,000 rpm for 10 mins at 4 ^o^C in TLA 120. To extract proteins, 1x Tris-glycine SDS sample guffer (Invitrogen, CA) were directly added to the pellets containing whole cells, mitochondria, or carbonate extracted membrane. Protein levels were determined using a Nanodrop (Nanodrop Technologies) and 20 µg of proteins were loaded into each well. We used 4-20% Tris-Glycine polyacrylamide gels (Invitrogen, CA). Gels were tranfered to PVDF membrane. After blocking with 5% non-fat dry milk in phosphate-buffered saline containing 0.1% Tween-20 (PBST), blots were probed with indicated primary antibodies, washed three times with PBST, incubated with secondary antibody conjugated with horse-radish peroxidase (HRP), and developed using chemiluminescence substrates and X-ray film. For Parkin experiments, cells were harvested and suspended in cell homogenization buffer (250 mM Sucrose, 20 mM Hepes-KOH, pH7.5, 1 mM EDTA, complete protease inhibitor [Roche]). Cells were homogenized by 20 passages through a 25-gauge needle with 1 ml syringe, on ice. The homogenates were separated into post-nuclear supernatant (PNS) by centrifugation, 1,000 xg for 5 min at 4 ˚C. Heavy membrane fraction was separated by spinning down supernatant from PNS separation at 3,000 xg for 10min at 4˚. To wash the heavy membrane fraction the pellet was resuspending with fresh buffer and spun back down two times. The supernatant from the previous fractionation was spun down at 16,000 xg for 15 min at 4˚ to collect the light membrane fraction. The remaining supernatant was the post-membrane fraction. Light membranes and heavy membranes were combined to form the membrane fraction (memb). 10 ug was loaded per ilane in PNS and the membrane fraction was loaded at 3 folds the PNS fraction.

**Quantification of Mitotracker Intensity in Mfn1/2^-/-^ MEFs.** Mfn1/2^-/-^ MEFs transfected with PINK1-YFP were pulsed with 50 nM Mitotracker Red (MTR) (molecular probes) for 10 minutes followed by washout for 5 minutes and fixation. A mid-plane image was obtained for each cell. MTR intensity and area was calculated for mitochondria with PINK1-YFP signal and mitochondria without PINK-YFP signal, respectively, in each cell, using ImageJ (NIH), by an experimenter blind to MTR intensity of those mitochondria. From these values an average MTR intensity/pixel/cell was calculated for PINK1-YFP positive and PINK-YFP negative mitochondria, respectively. The statistical significance of the difference in average MTR intensity/pixel/cell between PINK1 positive and PINK1 negative mitochondria was calculated using a paired t-test.

**Quantification of Colocalization in HeLa Cells Treated with Paraquat.** Cells were pulsed with 50 nM Mitotracker Red (MTR) (molecular probes) for 10 minutes followed by washout for 5 minutes and fixation. A mid-plane image was obtained for each cell. A ROI was drawn around the cell of interest and the pearson coefficient for the correlation in pixel intensity between two channels (e.g. PINK1-YFP and MTR) was calculated using the Wright Cell Imaging Facility (WCIF) plug-in for ImageJ.

**Measurement of Mitochondrial Mass.** Cells were washed with HBSS, trypsinized, and resuspended in HBSS. Cells were then incubated with 100nM Mitotracker Green (Molecular Probes) for 30 minutes, after which mean fluorescent intensity was measured on a FACS Diva (BD Biosciences) flow cytometer.

**Measurement of Mitochondrial Turnover.** For each sample, 2 X 10^5^ cells were plated a well of a 24 well plate. The cells were allowed to attach over night and the next day were incubated with 100 mM Mitotracker Green (MTG) in the presence 10 μM CCCP. Cells were washed three times in HBSS and then incubated in 10 μM CCCP for 0, 16, or 24 hrs. Cells were then washed once in HBSS, trypsinized, resuspended in HBSS and pipette into a well of a 96 well microtiter plate (Corning). Mean MTG fluoresce (485 nm/ 535 nm, 0.1 S) was measured using a Victor3 plate reader (PerkinElmer).
